# 3D hotspots of recurrent retroviral insertions reveal long-range interactions with cancer genes

**DOI:** 10.1038/ncomms7381

**Published:** 2015-02-27

**Authors:** Sepideh Babaei, Waseem Akhtar, Johann de Jong, Marcel Reinders, Jeroen de Ridder

**Affiliations:** 1 Delft Bioinformatics Lab, Faculty of Electrical Engineering Mathematics and Computer Science, Delft University of Technology, Mekelweg 4, 2628 CD Delft, The Netherlands; 2 Division of Molecular Genetics, The Netherlands Cancer Institute, Plesmanlaan 121, 1066 CX Amsterdam, The Netherlands; 3 Division of Molecular Carcinogenesis, The Netherlands Cancer Institute, Plesmanlaan 121, 1066 CX Amsterdam, The Netherlands

## Abstract

Genomically distal mutations can contribute to the deregulation of cancer genes by engaging in chromatin interactions. To study this, we overlay viral cancer-causing insertions obtained in a murine retroviral insertional mutagenesis screen with genome-wide chromatin conformation capture data. Here we find that insertions tend to cluster in 3D hotspots within the nucleus. The identified hotspots are significantly enriched for known cancer genes, and bear the expected characteristics of *bona fide* regulatory interactions, such as enrichment for transcription factor-binding sites. In addition, we observe a striking pattern of mutual exclusive integration. This is an indication that insertions in these loci target the same gene, either in their linear genomic vicinity or in their 3D spatial vicinity. Our findings shed new light on the repertoire of targets obtained from insertional mutagenesis screening and underline the importance of considering the genome as a 3D structure when studying effects of genomic perturbations.

## Abstract (web summary)

Retroviral insertional mutagenesis is used for identifying genes involved in the development of cancer. Here, the authors overlay cancer-causing insertions with genome-wide Hi-C data and find that retroviral elements tend to cluster in 3D hotspots.

## Graphical abstract

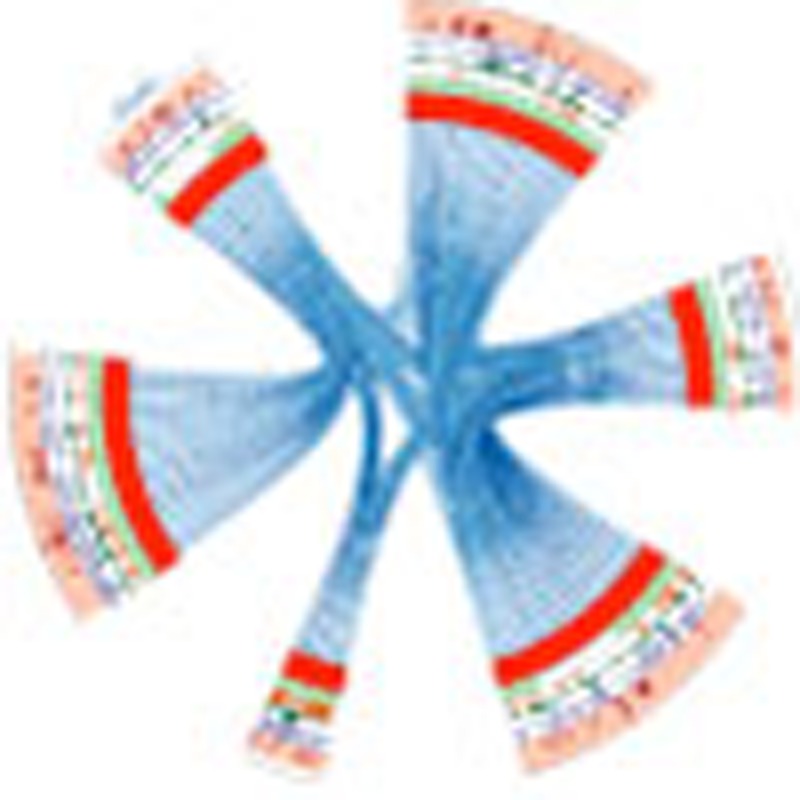

The three-dimensional (3D) organization of the genome appears to play an important role in carrying out the instructions encoded in its linear sequence. For instance, ample evidence suggests that the 3D conformation of chromosomes in the cell nucleus is an important factor in gene expression regulation^1^,^2^,^3^. This is because regulatory elements, such as enhancers, can act over large genomic distances by engaging in chromatin interactions with target genes, resulting in formation of chromatin loops^2^,^4^,^5^,^6^,^7^. An important example is given by the human beta-globin locus. In K562 cells, activation of the g-globin genes involves interactions between the locus control region and the activated genes, resulting from a large (~40 kb) chromatin loop^8^,^9^,^10^.

These observations are enabled by a technique called chromosome conformation capture or one of its variants, that is, 4C, 5C, Hi-C and ChIA-PET^11^,^12^,^13^. Coupled with next-generation sequencing technologies, the Hi-C approach was recently designed as an extension of the chromosome conformation capture method and allows detection of all pairwise physical interaction of DNA in the genome. As a result, the Hi-C contact matrix provides a comprehensive characterization of the chromatin conformation and insights into the 3D organizational features of the genome^14^.

Chromatin interaction studies also help to unravel the influence of 3D genome organization on complex genetic diseases such as cancer^15^,^16^,^17^,^18^. Some interesting findings pertaining to chromosomal alterations in cancer have already been made. In studies by Wijchers and de Laat^18^ and Engreitz *et al.*,^19^ for instance, the 3D organization of the genome was found to associate with the partner choice in chromosomal rearrangements. Fudenberg *et al.*^17^ showed that chromatin interactions are associated with the distribution of somatic copy number alterations. It was also reported that overexpression of oncogenic transcription factors is associated with 3D organization of the genome^15^.

The variability of chromatin interactions between cell types is mostly confined to local interactions^20^,^21^, while long-range interactions are relatively well conserved and stable^20^. This demonstrates that different cell types share a common global architecture of their chromosomes. Similar observations were made in a study of domains interacting with the nuclear lamina; Peric-Hupkes *et al.*^22^ and Meuleman *et al.*^23^ observed that the vast majority of lamina-associated domains are invariant among the four different cell types.

Here we study the effect of long-range chromatin interactions on co-localization of viral integrations in mouse cancer genomes. This is achieved by overlaying Hi-C data obtained in mouse embryonic stem (ES) cells and cortex cells, with cancer-causing insertional mutations obtained in murine lymphoma and leukaemia. Rather than investigating the landscape of translocations and copy number variations we aim to delineate the repertoire of target genes affected by insertional mutations, while taking into account the 3D conformation of the genome.

Retroviral insertional mutagenesis (IM) is a forward genetic screening technique for identifying genes involved in the development of cancer, and is based on the ability of retroviruses to insert their DNA in the genome of a host cell. Since the viral long-terminal repeats (LTRs), located at either end of the provirus, contain strong enhancer sequences, these insertions can lead to the deregulation of nearby genes^24^. Therefore, cells carrying insertions near cancer genes may have a selective advantage compared with cells without the insertion, resulting in clonal outgrowth. To identify these cancer genes, retroviral IM screens rely on the identification of clusters of insertions, that is, genomic regions that harbour recurrent insertions in multiple independent tumours. When such clusters are statistically significant, they are referred to as common insertion sites (CISs)^25^,^26^. CISs frequently arise near known human cancer genes and can thus be used to identify novel candidate cancer genes^26^,^27^.

CISs are defined as clusters of insertions along the linear genome without consideration of genome organization. However, long-range chromatin interactions might allow insertions to contribute to deregulation of genomically distal genes. As a result, insertions that deregulate the same gene may be found dispersed across multiple linearly distal but spatially proximal loci (Fig. 1). Therefore, it is important to consider the insertion clusters (ICs) in the context of their 3D arrangement.

Importantly, if this hypothesis is correct, the current practice of CIS calling and searching for nearby putative cancer genes based only on the linear genome is inadequate. More specifically, it has two important limitations. First, the insertion signal is diluted, since a cluster of insertions that affect the same gene is split across multiple distal loci. Consequently, many truly causal ICs may not reach the required significance threshold to be called a CIS. This is especially problematic in small sample sizes. Second, the genes in the genomic vicinity of the ICs are not necessarily the actual target genes. As a result, CISs that exert their effect by chromatin looping can be left without a clear target or even associated to wrong target genes.

To investigate this phenomenon, we assess whether ICs are in spatial proximity more frequently than expected by chance. In the following, we demonstrate that, in addition to the well-characterized clustering of insertions along the linear genome, insertions also co-localize according to the 3D conformation of the genome. This is important for determining the putative targets of insertions. Consequently, our spatial clustering approach identifies additional loci with putative cancer genes and improves the identification of putative target genes of many other ICs in retroviral IM screens. In addition, it provides new clues as to how long-range chromatin interactions are involved in (viral) enhancer activity.

## Results

### Data

Retroviral IM data were acquired from Mutapedia ( http://mutapedia.nki.nl). In total, this data set contains 19,997 viral insertion sites across 933 murine tumours of various genetic backgrounds. These tumours developed in a range of tissues, predominantly in the thymus, spleen and lymph node, and contained a mixture of B and T cells^26^.

To determine long-range spatial interactions between insertion sites, we integrated the IM data with publicly available Hi-C data, collected from mouse ES cells and cortex cells^20^. Experimental biases in the Hi-C data, such as guanine–cytosine content of trimmed ligation junctions and distance between restriction sites, were eliminated using the probabilistic model described by Yaffe and Tanay^28^. Only the intra-chromosomal Hi-C data were used. As a result, we obtained one Hi-C contact map that describes the ligation frequencies between pairs of bins for each chromosome. In addition to the original 40 kb resolution, we generate contact maps at 200 kb resolution by summing five consecutive bins. We eliminated the genomic distance bias that arises owing to preferential ligation between genomically proximal bins (Supplementary Fig. 1), using a rank-based normalization method that we developed for this purpose (see Methods).

### Insertion sites occur often in open chromatin compartments

**Figure 2:**
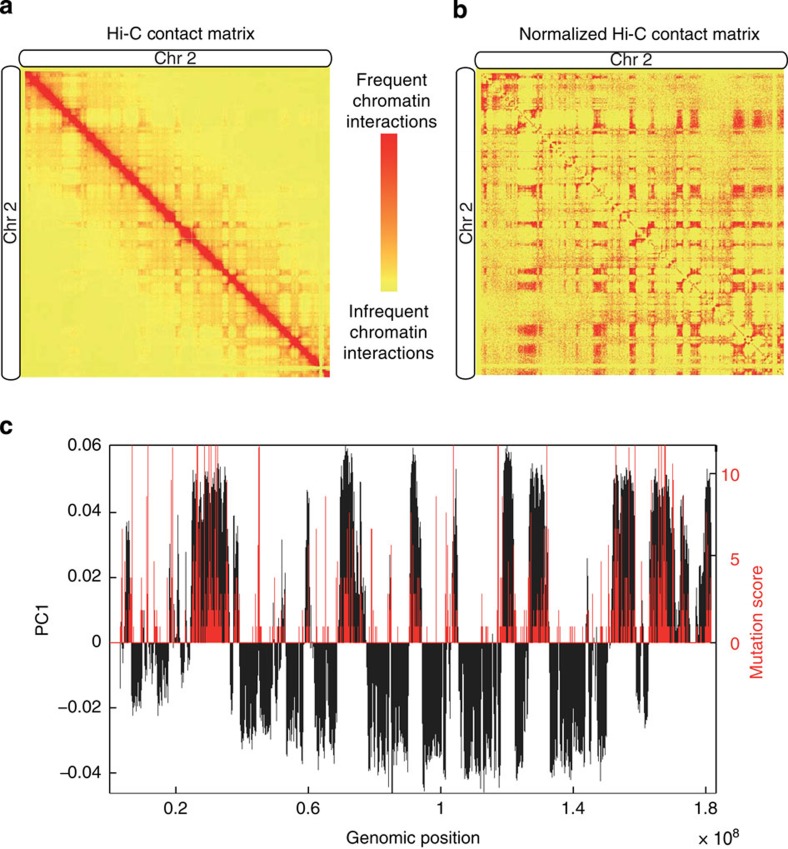
Normalized Hi-C contact matrix. (**a**) Hi-C contact matrix of chromosome 2. (**b**) Rank-normalized Hi-C contact matrix. (**c**) The insertion count per bin on each chromosome together with the value of the first principle component (PC1) of the correlation matrix of the normalized Hi-C contacts (at 200 kb resolution). A positive or negative value of PC1 indicates open or closed chromatin compartments, respectively.

After rank-based normalization, the first observation from the Hi-C contact maps is its plaid pattern as shown in Fig. 2b. Such patterns were previously described by Lieberman-Aiden *et al.*^14^, who showed that it points to a two-compartment model of chromatin. In particular, they reported that the first principal component (PC) of the normalized Hi-C matrix captures the distinction between open and closed chromatin compartments^14^,^21^,^29^.

To investigate the relation between mutations and chromatin compartments, we overlaid the insertion sites with the first PC of the normalized Hi-C contact map for each chromosome (Supplementary Fig. 2). Figure 2c shows the insertion count for 200-kb bins on chromosome 2, together with the value of the first PC. There is a strong correlation (Pearson correlation; *P* value=10^−10^) between open chromatin and the presence of insertions. Such strong correlation is observed for all chromosomes (least significant *P* value=0.01; Supplementary Fig. 3). This suggests that insertions preferentially occur in open chromatin (Supplementary Fig. 4), which is more accessible and associated with higher gene density and higher messenger RNA expression^14^,^30^. While perhaps not unexpected and previously observed^31^, this finding does confirm that overlaying IM and Hi-C data can yield useful results even though they are not obtained from the same cells.

### Recurrently inserted bins are spatially co-localized

**Figure 3:**
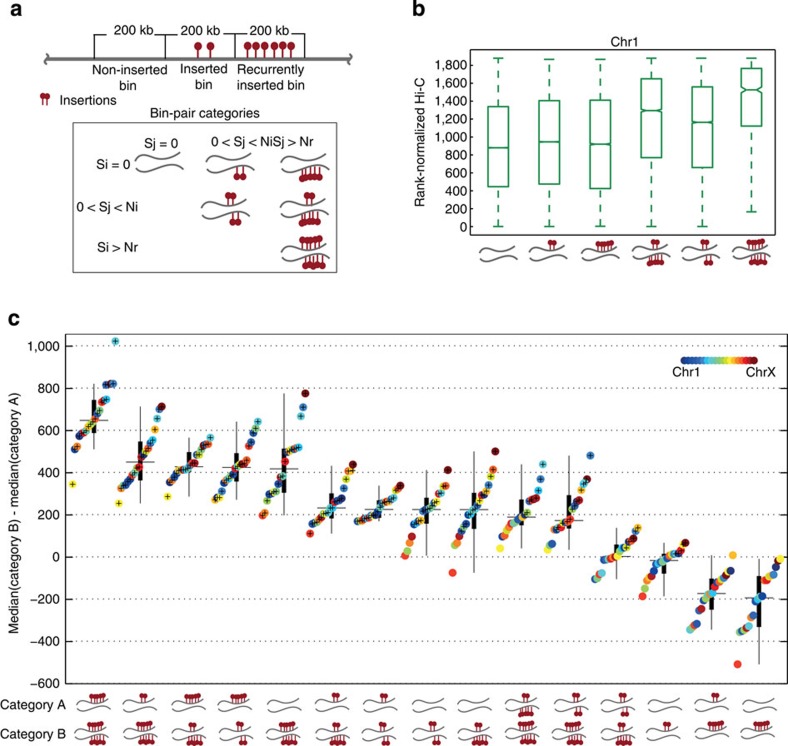
Comparison of Hi-C contact score distributions. (**a**) Bin-pair categories based on the insertion count (*S*) per 200-kb bin. Bins are divided into non-inserted (NI; *S*_*i*_=0), inserted (I; 0<*S*_*i*_≤*N*_*m*_) or recurrently inserted (RI; *S*_*i*_>*N*_*r*_) categories. (**b**) Distribution of rank-normalized Hi-C interactions for six bin-pair categories for chromosome 1. The other chromosomes are plotted in Supplementary Fig. 5. We select *N*_*m*_=2 and *N*_*r*_=5 (see Supplementary Fig. 4). (**c**) Boxplot comparing Hi-C interaction of the six bin-pair categories including 15 unique combinations. This is done for all chromosomes, that is, each box represents 20 values. The *y* axis represents the difference between the median Hi-C score of bin-pair category A and bin-pair category B. A starred circle indicates a significant difference between medians for that chromosome (Wilcoxon rank-sum test; *P* value<10^−10^). The bin-pair categories that are compared are schematically illustrated under each box. Boxes are sorted based on their medians.

To examine whether insertion sites are co-localized in the context of the spatial organization of the genome, we determined Hi-C contact scores between three categories of inserted bins (Fig. 3a). We distinguished between bins that are non-inserted, inserted or recurrently inserted. The first category represents regions that are unlikely to cause cancer on integration or cannot be integrated altogether. The second category represents regions in the genome that are accessible to integration, but without sufficient evidence that insertions in these bins are causing cancer, that is, these regions contain mostly background insertions. The latter category represents regions in the genome that are likely to be under selective pressure (akin to a CIS), and are thus likely to be cancer causing. For this reason, we used 200-kb bins, which are approximately the average size of CISs^26^, for this analysis. We compared the Hi-C scores obtained from the 200-kb contact maps for all combinations of categories (in total six combinations, Fig. 3b) for all chromosomes (Supplementary Fig. 5). Results for different bin sizes are provided in Supplementary Fig. 6, showing that 200 kb is indeed the most discriminating resolution.

Figure 3c summarizes the results when comparing the medians of Hi-C contact score distributions for all combinations of bin-pair categories (in total 15 unique combinations). It shows that bin pairs of which both bins harbour insertions (either recurrent or just a few) have stronger Hi-C contact scores than bin pairs where only one or none of the bins harbour insertions (Wilcoxon rank-sum test; *P* value<10^−10^). This implies that inserted regions in the mouse genome tend to be co-located in 3D hotspots.

The most significant differences, however, are observed when comparing co-localization of bin pairs of which both bins are recurrently inserted with bin pairs of which at least one bin does not harbour any insertions (two left most boxes in Fig. 3c). Since recurrence of insertions across multiple independent tumours is an indication of selection, this suggests that co-localization of recurrently inserted loci arises—at least to some extent—by selective pressure.

Murine leukaemia virus integration is known to be non-uniform across the genome. Recently, it has been found that integration of murine leukaemia virus is promoted by BET proteins at transcription start sites (TSSs)^32^. Therefore, we investigated spatial association for the subset of bins that contain at least one TSS. Supplementary Figure 7a shows that the results obtained are highly similar, indicating that local clustering around TSSs does not impact our findings. Moreover, insertion sites are more frequently found in domains of open chromatin. We therefore also analysed spatial association between the subsets of loci that are located in the same (open or closed) chromatin compartments. Comparing the effect size of these hypothesis tests (Supplementary Fig. 7b) shows that the observed co-localizations are somewhat reduced but remain significant. We also repeated these analyses using Hi-C data obtained from cortex cells (Supplementary Figs 8 and 9). The results are very similar to those obtained with ES cell Hi-C data, demonstrating that the results are insensitive to cell type. Finally, we observe that randomizing the insertion locations uniformly across the genome or in bins with at least one TSS destroys the observed association (Supplementary Fig. 10a,b).

In an orthogonal analysis (Supplementary Fig. 11), we considered the insertion scores of the nearest spatial neighbour bin of an inserted bin (that is, the bin that has the maximum Hi-C contact with the bin of interest). For most chromosomes, we observe highly significant insertion scores for nearest spatial neighbour bins, corroborating our observation that inserted bins are spatially co-located.

### Detecting co-localized ICs

**Figure 4:**
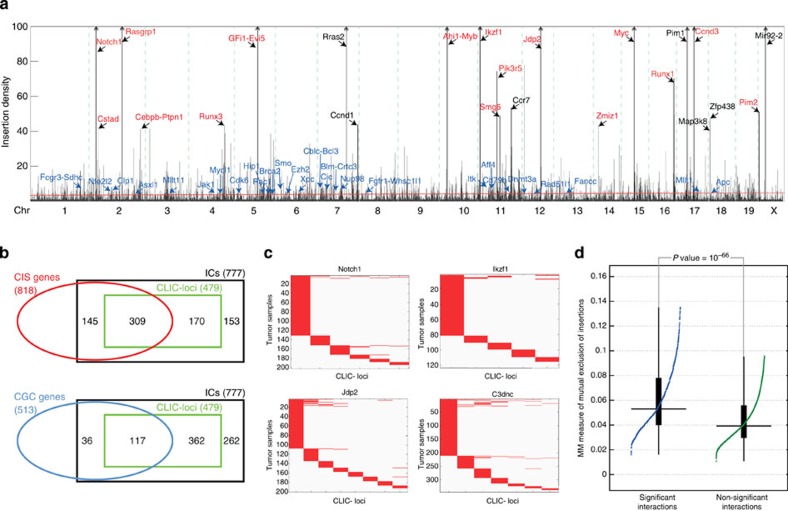
Properties of CLICs and CLIC loci. (**a**) Kernel-smoothed insertion count (80-kb Gaussian kernel) along the mouse genome. ICs are identified as peaks. The red line shows the median peak height (5.2), below which insertions are considered to be background insertions, that is, not contributing to tumor development. Genes associated to CISs with peak height >40 are shown (23 CISs). Among the top CISs, 19 genes that are also detected by our CLIC analysis are indicated in red. The 31 known cancer genes that are discovered as CLIC loci are indicated in blue. The insertion density in the genomic vicinity of these genes is not sufficient to be detected as CIS genes. (**b**) Venn diagram indicating overlap of CLIC loci with CIS and Cancer Gene Census (CGC) genes. (**c**) Patterns of mutual exclusive insertions in CLIC loci that form CLICs with the *Notch1*, *Ikzf1*, *Jdp2* and *Ccnd3* loci. A red colour indicates that an insertion occurred in the corresponding sample (*y* axis) and CLIC locus (*x* axis) (**d**) Boxplots of Mean-Manhattan distances between significant CLIC loci (that is, actual CLICs) and non-significant combinations of CLIC loci. This distance is defined as the fraction of samples with one of two loci inserted and captures mutual exclusivity. CLICs exhibit a highly significant (Wilcoxon rank-sum test; *P* value=10^−66^) pattern of mutual exclusive insertions.

We next established which pairs of inserted loci in the mouse genome are co-localized in 3D hotspots. To this end, we first defined ICs by selecting peaks in the kernel-smoothed insertion count (Fig. 4a) along the linear genome 26. We selected the 777 high-scoring ICs with a peak height exceeding the median peak height. This ensured that also ICs that did not reach the traditional CIS threshold (which is much higher) are included in the analysis. For this reason, of these 777 clusters, only a subset (454) overlaps with one or more putative CIS-target genes previously identified in ref. 26.

To determine chromatin interactions between ICs, we compared the distribution of Hi-C values between the high-scoring ICs with the distribution of Hi-C values between all bins at a comparable genomic distance. In spirit, this is similar to the normalization used before and described in the Methods section. Note that, as in this analysis the insertion data is not binned, we can use the Hi-C data at its original resolution of 40 kb. A Wilcoxon rank-sum test was used to determine whether two ICs are more frequently spatially co-localized than expected by chance (see Methods for details). Only the 17,102 possible intra-chromosomal combinations of high-scoring ICs were considered. A pair of ICs with significant chromatin interactions is called a co-localized IC (CLIC). The two loci that constitute a CLIC will henceforth be referred to as CLIC loci. Note that each CLIC locus corresponds to an IC, but that an IC is only a CLIC locus in case they engage in one or more significant chromatin interactions, that is, when they are part of one or more CLICs.

### Major CIS genes are involved in CLICs

We found 874 CLICs, encompassing a total of 479 unique CLIC loci. An overview of the CLICs is given in Supplementary Fig. 12 and Supplementary Table 1. As expected, the majority (309 or 65%) of these loci encompass genes previously identified in ref. 26. This overlap is significantly higher (Fisher’s exact test, *P* value=1.4 × 10^−5^) than the overlap observed for ICs that did not form CLICs (Fig. 4b). Moreover, we also determined significant interactions among pairs of low-scoring ICs (that is, ICs not exceeding the median peak height; 130,134 possible intra-chromosomal IC pairs). Of these ICs, only 308 of these reach the significance threshold, which is significantly less than interactions discovered among high-scoring ICs (Fisher’s exact *P* value≪10^−10^). This supports our previous observation that the 3D co-localization arises more frequently between recurrently inserted loci and is, at least to some extent, driven by selective pressure. Figure 4a shows the 16 CLIC loci that coincide with 19 major CIS genes (those with peak height exceeding 40).

### Insertions in CLICs exhibit a pattern of mutual exclusion

We observed that insertions in CLICs exhibit a distinct pattern of mutual exclusion, that is, insertions are unlikely to co-occur in the two loci in the same sample. This is exemplified in Fig. 4c, which shows the insertion profiles across the samples for four CLICs with a strong mutually exclusive insertion pattern. These CLICs contain *Notch1*, *Ikzf1*, *Jdp2* and *Ccnd3*, all of which are top CIS genes. Typically, mutually exclusive mutations provide evidence that the mutated loci are functionally linked in a common biological pathway as alteration of a second locus within the same pathway offers no further selective advantage^33^. In case of insertions in CLICs, however, a more likely explanation is that insertions in two CLIC loci target the same gene, either in their linear genomic vicinity or in their 3D spatial vicinity.

Mutual exclusion is apparent for many more CLICs. To characterize this, we scored mutual exclusion for two loci by the Mean-Manhattan distance, defined as the fraction of samples with one of two loci inserted. The distribution of distances for CLICs and all non-significant combinations of high-scoring ICs are compared in Fig. 4d. We found that CLICs exhibit a highly significant (Wilcoxon rank-sum test; *P* value=10^−66^) pattern of mutual exclusion, supporting the hypothesis that insertions in both loci target the same gene.

### CLIC loci coincide with cancer genes

We determined the occurrence of known cancer genes within CLIC loci. We used the list obtained from Cancer Gene Census (accessed: Jan 2014) for this purpose^34^. A substantial number of CLIC loci (117, 25%) overlap with previously identified cancer genes (Fig. 4b; Supplementary Table 2). This is significantly higher (Fisher’s exact test, *P* value=1.8 × 10^−5^) than the overlap observed for high-scoring ICs that do not form CLICs. Thus, insertions in regions engaged in 3D interactions are more likely to deregulate genes causing cancer.

Of the known cancer genes within a CLIC locus, 31 (in 27 CLIC loci) were not among the list of CIS genes (Fig. 4a). This shows that, even though these genes are not identified in a traditional CIS analysis, they can be identified by taking into account chromatin interaction with a second inserted locus.

Among these genes are *Brca2*, *Fancc*, *Apc* and *Jak1*. For all of these genes, the literature provides evidence that suggests a role in the haematopoietic neoplasms under study. For instance, while germline mutations in human BRCA2 are known to predispose for breast and ovarian cancer, recently, evidence was presented suggesting that mutations in the BRCA pathway also significantly increase the risk for certain leukaemias and lymphomas^35^. Moreover, mutations in BRCA2 are also known to be responsible for Fancomi anaemia (FA)^36^. Patients suffering from FA are much more likely to develop leukaemia. Of note, another FA gene, *FANCC*, was also among the list of CLIC genes. Furthermore, *APC*, a gene with a role in a plethora of cancers has also been implicated in promoting leukaemia^37^. Finally, JAK1, a tyrosine kinase, was found to be recurrently mutated in acute leukaemias^38^. Taken together, these findings suggest that insertions in clusters that are not identified by a CIS may point to *bona fide* putative cancer genes. These cancer genes can only be discovered by aggregating evidence across multiple loci co-localized by 3D conformation.

### CLIC loci link distal insertions to known cancer genes

The majority of CLIC loci (362, 76%) do not overlap a known cancer gene. However, 54% of these CLIC loci form a CLIC with a CLIC locus that does harbour a known cancer gene. This leaves only 166 (35%) CLIC loci without a known cancer gene (corresponding to 463 (53%) CLICs for which none of the CLIC loci overlap to any known cancer genes). This number is significantly lower when compared with a control set of high-scoring IC pairs that do not participate in a CLIC (Fisher’s exact test, *P* value=4 × 10^−8^). Taken together, this suggests that insertions in these CLIC loci may be involved in deregulation of a known cancer gene by long-range chromatin interactions.

**Figure 5:**
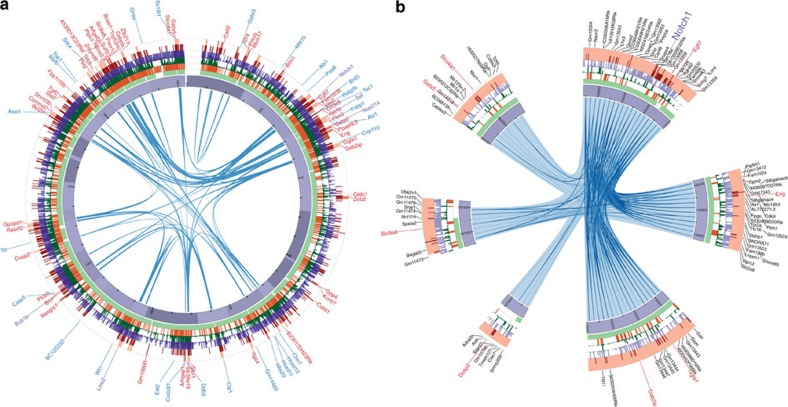
CLIC loci on chromosome 2. (**a**) Circos plot of chromosome 2. The purple track indicates the chromosome. Light green, orange, dark green, purple and red tracks indicate topologically associated domains (TADs), enhancers, DNase I hypersensitive sites (DHSs), transcription factor-binding sites (TFBSs; for cMyc, CTCF, Taf3, Zfx and Mcaf1), and insertion sites, respectively. Tick marks appear every 5 Mb on the chromosome. The ICs are indicated by light red. Links indicate significant Hi-C contacts between ICs. CIS and CGC genes are indicated by red and blue, respectively. (**b**) CLICs containing the *Notch1* locus. Tick marks appear every 100 kb on the chromosome. Genes in each CLIC locus are indicated by black.

Figure 5 shows an example of CLICs that link insertions to a distal cancer gene. These CLICs encompass six CLIC loci on chromosome 2, one of which overlaps the *Notch1* gene, a common target in retroviral IM screens^39^. In this screen, *Notch1* harbours insertions in 130 independent tumour samples. It is a member of the family of NOTCH receptors that operate both as recipients of extracellular signals at the cell surface and as transcription factors (TFs) regulating gene expression in the nucleus. Notch signalling plays an important role in haematopoietic neoplasms, including leukaemia and lymphoma^40^.

The five CLIC loci that co-localize with the Notch1 locus collectively harbour insertions in 63 independent tumour samples. While it is possible that insertions in these CLIC loci target genes in their direct genomic vicinity, another explanation is that insertions affect *Notch1* across a large genomic distance by long-range chromatin interactions. This is supported by the observation that insertions across the six CLIC loci exhibit a distinct pattern of mutual exclusion (Fig. 4c). Notably, this observation casts doubt on the previously reported CIS-target genes in these five loci, demonstrating the importance of taking into account chromatin interactions in associating ICs to putative target genes.

### CLIC loci interacting with cancer genes enrich for TFBSs

For CLICs for which one of the two loci contains a known cancer gene (say locus A), we searched for enrichment of TF-binding sites (TFBSs) in the locus without the known cancer gene (locus B). Finding such TFBSs may be an indication that locus B contains regulatory elements that are involved in the regulation of the cancer gene in locus A and that insertion of a viral LTR in that locus can boost this regulatory interaction activating the cancer gene at locus A.

To this end, we used previously published chromatin immunoprecipitation-sequencing data for 50 TFs in mouse ES cells (collected and preprocessed in ref. 31). Model-based Analysis of ChIP-Seq (MACS) was used for peak calling^41^ and a *P* value cutoff of 10^−5^ was employed to determine the TFBSs. A CLIC locus is called a TFBS carrier if it contains more TFBS peaks than the median number of peaks across all ICs. Note that the number of peaks in an IC was normalized by the size of the ICs.

We found that among the 337 CLICs that overlapped a cancer gene in locus A, 230 are TF carriers in locus B. This is significantly more than in a control set of high-scoring IC pairs that do not participate in a CLIC (Fisher’s exact test *P* value=2.6 × 10^−9^). This implies that known cancer genes frequently co-localize with distal loci that are enriched for regulatory elements that aid viral insertions at those distal loci to influence the activity of cancer genes.

### Insertions in CLIC loci affect co-localized gene expression

The viral enhancer sequences of insertions can deregulate expression of genes nearby in the linear genome. If insertions can target linearly distal genes by long-range chromatin interactions, a similar deregulation should be observed for genes in CLICs, that is, for genes co-localized with distal insertions.

To test this hypothesis, we analysed gene expression data available for a subset of 99 samples^42^,^43^. For each gene *g*_*i*_ that resides in a CLIC locus, say CLIC locus A, we quantify the activating or repressing effect of insertions on the expression of *g*_*i*_. To do this, the 99 samples are divided into two groups based on the presence of insertions in linear proximity to gene *g*_*i*_, that is, based on insertions in CLIC locus A. We then quantify whether these insertions influence the expression of gene *g*_*i*_ by calculating a two-sample *t*-statistic between samples with and without insertions, resulting in score 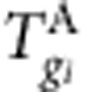. To investigate the long-range effect, we also divided the 99 samples in two groups based on the presence of insertions in 3D spatial proximity to gene *g*_*i*_, that is, based on whether there are insertions in CLIC locus A or in the second locus of the CLIC, CLIC locus B. Again, the difference in expression of gene *g*_*i*_ between samples with and without insertions is quantified with a two-sample *t*-statistic, resulting in score 
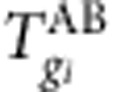
.

If distal insertions contribute to the deregulation of genes, we expect to see an increased activating or repressing effect when considering all 3D co-located insertions rather than only the linearly proximal insertions. We confirmed that this is the case by comparing *T*^AB^−*T*^A^ (if *T*^A^>0 otherwise we compared *T*^A^−*T*^AB^) for all genes in CLICs with genes in a control set of high-scoring IC pairs that do not participate in a CLIC. This comparison is statistically significant (one-sided *t*-test, *P* value=2 × 10^−5^; Supplementary Fig. 13) and demonstrates that insertions can have a measurable and significant effect on expression of co-localized genes.

We moreover find that 40% (409 among 1,019) of genes located in the 28 CLIC loci that harbour insertions in more than five samples displayed increased spatial association scores (sign(*T*^A^)(*T*^AB^−*T*^A^)>0). These include prominent insertional target genes such as *Myb*, *Gfi1* and *Ccnd2* (Supplementary Table 3). Interestingly, recent insight into *Myb* transcriptional regulation suggests that long-range chromatin interactions control Myb proto-oncogene transcription^44^.

## Discussion

In this study, we have presented a comprehensive analysis to explore the long-range chromatin interactions between clusters of cancer-causing retroviral insertions in the mouse genome. We found a significant co-localization of ICs that is markedly more pronounced for regions with insertions across multiple independent tumours. This is an indication that 3D hotspots of recurrent mutations are under selective pressure. To identify such hotspots, we defined CLICs, that is, pairs of loci with insertions that engage in frequent chromatin interactions. We found that insertions in CLICs are mutually exclusive, which is indicative of a shared target gene. Moreover, we observe an increased effect on gene expression by taking into account linearly distal yet spatially co-located insertions, supporting the hypothesis that insertions can act on targets by long-range chromatin interactions. About half of the CLICs are associated with known cancer genes, and are enriched for regulatory elements.

Combined, our analyses suggest that long-range interactions, which arise as a result of the 3D organization of the genome, play an important role in determining the genes that are targeted by insertions. This is in line with a recent comprehensive survey of 3D contacts across different developmental contexts, which show that in many cases the long-range spatial contacts between promoters and enhancers are stable across cell types including in cellular contexts where the promoter is not active (unproductive or poised contacts)^45^. Therefore, introduction of strong transcription-promoting elements, encoded in the LTRs of a virus, by IM might transform unproductive (poised) 3D connections into productive ones, leading to activation of the target promoter. The traditional definition of a CIS and the subsequent search for the most likely target gene in its genomic vicinity, may therefore lack power to identify true cancer genes. We found, for instance that 126 (~30%) of the previously identified CIS loci are co-localized with known cancer genes by long-range chromatin interaction (Supplementary Table 2). These known cancer genes might be more likely target genes than the originally reported genes in the direct genomic neighbourhood of the insertions. While we show that an integrative analysis of insertional mutations with Hi-C data from a different source yields valuable results, such results do not unequivocally establish a relation between mutations and their targets in the same cells. Ultimately, it is, therefore, desirable to establish target genes based on the chromatin conformation capture data that was derived from the same source as the mutations. If those data become available, our approach provides the necessary framework to integrate these data.

Considering the 3D organization of the genome in the analysis of IM, data can also reveal novel target genes. For instance, based on our CLICs, we retrieve 31 known cancer genes that were not identified by a traditional CIS analysis. Moreover, we identified 16 CLIC loci that did not overlap nor co-localize with any previously identified CIS genes or known cancer genes. Ingenuity Pathway Analysis (IngenuitySystems, www.ingenuity.com) revealed that of the 83 genes in these loci, 42 can be linked to cancer (enrichment *P* value=1.8 × 10^−5^). Among these are *Stat1*, *Stat2*, *Il23r* and *Il12rb2*, all of which are members of the JAK–STAT signalling pathway. This pathway is known to be deregulated in haematopoietic malignancies^46^. Another gene with strong precedent within the literature is *Cpt1a*, which recently has been identified as member of a 24 gene prognostic signature for acute myeloid leukaemia^47^. Collectively, these results demonstrate that, by incorporating chromatin interaction data in the analysis, novel target genes may be identified that were missed in the conventional analysis of the screen.

Retroviral insertions also play an important role in causing certain human cancers such as hepatocellular carcinoma^48^. We have applied our methods to a set of hepatitis B virus insertion sites, which are acquired from two studies of hepatocellular carcinoma patients^49^,^50^. In total, the data consists of 550 insertion sites across 50 samples. Despite the limited data set size, we found significant co-localization of insertion sites in chromosome 2, 4, 11, 15 and 16 (Wilcoxon rank-sum test; *P* value<10^−5^; Supplementary Fig. 14), which potentially points to shared target genes for integrations at these distal loci. Moreover, retroviral integration is at the basis of gene therapy where retroviruses are used to deliver transgenes into a host genome^51^,^52^. For this reason, our results are important for gene therapy treatment of, for example, X-linked adrenoleukodystrophy and severe combined immune deficiency^52^,^53^ as long-range chromatin interactions might explain the unintentional insertional activation of oncogenes, which has been observed as an adverse effect of treatment with retroviral vectors^54^.

More generally, our findings have important implications for the study of non-viral mutations that affect enhancer activity in human tumours. Such non-coding mutations are currently being identified at increasing rate owing to whole-cancer genome sequencing and increasing scale of genome-wide association studies. Recently, evidence is emerging that genome-wide association study hits can be functionally connected to distal genes. One prominent example is provided by the variants within *FTO* that are now convincingly linked to the regulation of *IRX3* expression by direct long-range chromatin looping^46^. Our approach will aid in delineating the role of genome conformation in target gene selection, as well as help to determine which genes are affected by these mutations.

Taken together, we find that long-range chromatin interactions of tumour-driving insertions are prevalent. This sheds new light on the repertoire of targets obtained from IM screening and underlines the importance of considering the genome as a 3D structure when studying effects of genomic perturbations.

## Methods

### Binning of Hi-C and insertion data

We have used Hi-C data at two resolutions: the original resolution of 40 kb and a decreased resolution of 200 kb. The insertion data were only binned at 200 kb. This bin width corresponds to the average size of a CIS, thus ensuring that the classification of bins into the ‘recurrent’ class is done at an appropriate resolution. The 200-kb bin width is used to establish a genome-wide overview of co-localization (Fig. 3). For detecting CLICS, insertions are clustered using kernel smoothing (see below) and overlaid with the 40 kb resolution Hi-C data (Figs 4 and 5).

### Detecting ICs

ICs are determined as the local extrema (peaks) in the kernel-smoothed insertion count *f*(*x*), obtained by Gaussian kernel convolution (GKC): 
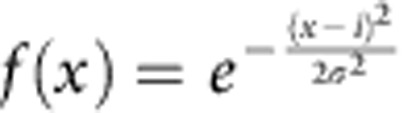
. The size of an IC is controlled by the width of the Gaussian kernel *σ* and depends on the local density of insertion sites. In our analysis, we set 2*σ* to 80 kb. To deal with the fact that the Hi-C data is binned, the IC boundaries are aligned to the nearest bin boundary. This results in clusters spanning between 40 and 800 kb, which is within the range of minimum and maximum size of CISs^26^, and often contain multiple genes. We kept ICs with a peak height exceeding 5.23, the genome-wide median of *f*(*x*), retaining only those ICs for which some evidence for selection is observed. Note that this threshold is lower than the threshold required for statistical significance (that is, the threshold for calling CISs)^26^.

### Rank-based normalization

Two regions with a small genomic distance have higher numbers of chromatin interactions, independent of the actual 3D organization of the genome. Therefore, several studies have defined normalized Hi-C contact maps by accounting for the spatial bias in the Hi-C signal^14^,^16^. However, these methods assume that Hi-C contacts between regions with the same genomic distance are normally distributed, which is clearly not the case. Moreover, the distributions are quite variable for bin pairs at different genomic distances. Therefore, we applied a rank-based normalization technique to eliminate the effect of the genomic distance on intra-chromosomal Hi-C contacts.

In this rank-based approach, each Hi-C score is replaced by its relative rank compared with Hi-C scores between bins with a similar distance in the genome. Consider *h*_*ij*_, the Hi-C contacts between bin *i* and *j* with genomic distance of *d*. The normalized score 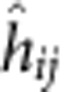 is defined as its rank in the vector *H*^*d*^, where *H*^*d*^ is the *k*^th^ superdiagonal of *H* (with 
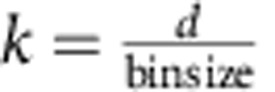
; see also Supplementary Fig. 15). This vector thus contains the Hi-C scores between all bin pairs that have the same genomic distance *d*. If any two values in *H*^*d*^ are tied, their average rank is used.

Evidently, the length of *H*^*d*^ decreases as the genomic distance increases. Therefore, these vectors are extended to have an equal length *L*, by including the appropriate number of elements from the *k*−*n* and *k*+*n* superdiagonals. Note that for large *k*, a substantial number of elements from neighbouring superdiagonals are included. This is appropriate as we observed that the distributions of *H*^*d*^ are more similar for large *d*, and can thus be pooled. We set *L* to be equal to twice the number of bins on chromosome 1, the longest chromosome.

Using rank-based normalization, we observed a similar plaid pattern (Supplementary Fig. 1), as obtained using the normalization method in ref. 14 (Supplementary Fig. 16). Moreover, we repeated the analysis resulting in Fig. 3c using the normalization method in ref. 14. Similar, yet less pronounced, results are observed (Supplementary Fig. 17).

### Detecting CLICs

We tested co-localization for all combinations of ICs within the same chromosome (17,102 possible IC pairs). The co-localization of two ICs, *IC*_*i*_ and *IC*_*j*_, with size of *n* and *m* bins, respectively, is described by the *n* × *m* matrix of Hi-C scores. To test whether these values are higher than expected by chance, we compared their distribution (positive distribution) to the distribution of bins with approximately the same genomic distance (negative distribution), using a one-tailed Wilcoxon rank-sum test. To guarantee that there is a sufficient number of values in the positive distribution, we set the minimum size of an IC to three bins so that each positive distribution consists of at least 3 × 3=9 points. To this end, ICs with size of less than three bins are expanded to cover one more bin on both sides.

The negative distribution is generated by concatenating Hi-C scores between bins with genomic distances in the range of *δ*=(*d*_min_, *d*_max_), where *d*_min_ and *d*_max_ are defined as the minimum and maximum genomic distance between *IC*_*i*_ and *IC*_*j*_, respectively (Supplementary Fig. 15). The range of *δ* is increased to ensure the negative distribution contains at least *L* Hi-C scores.

The discovery set of CLICs is defined as the IC pairs with Hi-C interactions exceeding the significance level *α*=10^−5^. Correction for multiple testing is achieved by controlling family wise error rate using the Holm’s procedure^55^ for 17,102 tests.

### Expression analysis

We used Illumina MouseWG-6 v2.0 expression measurements on set of 99 samples, a subset of the 933 samples used here^42^,^43^. Gene expression data were normalized using variance-stabilizing transformation and robust spline normalization^56^. Illumina probes with no corresponding RefSeq ID were discarded, leaving 45,281 measurements. Averaging over probes mapping to the same RefSeq ID resulted in expression values for 19,010 genes.

## Author contributions

S.B. implemented the methodologies and analysed the data. J.d.R. and M.R. conceived the experiments and mentored the project. J.d.R. and S.B. wrote the manuscript with contributions from M.R. J.d.J. performed the ChIP-Seq analysis. W.A. supervised the biological interpretation. All authors revised, discussed and amended the manuscript and approved its final version.

## Additional information

**How to cite this article:** Babaei, S. *et al.* 3D hotspots of recurrent retroviral insertions reveal long-range interactions with cancer genes. *Nat. Commun.* 6:6381 doi: 10.1038/ncomms7381 (2015).

## Supplementary Material

Supplementary Information Supplementary Figures 1-17

Supplementary Data 1 List of Co-localized Insertion Clusters (CLICs)

Supplementary Data 2 List of CLIC-loci

Supplementary Data 3 List of genes with increased spatial association scores for two thresholds

## Figures and Tables

**Figure 1:**
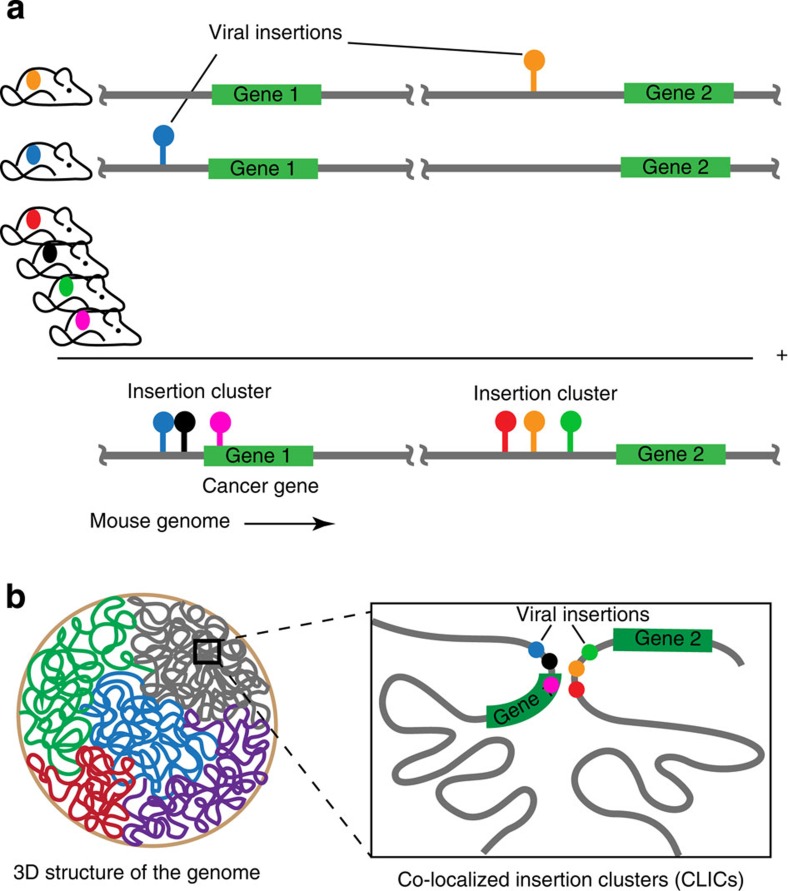
Illustration of insertions in the 1D and 3D genome. (**a**) A cancer candidate gene is traditionally determined by searching for a gene that harbours frequent mutations in its genomic vicinity across multiple independent tumours. (**b**) Genomically distal insertions can affect gene activity by 3D conformation of the genome. For instance, insertions that deregulate gene 1 may be dispersed across a pair of co-localized insertion clusters (CLICs) that engage in frequent chromatin interactions. As a consequence, the mutation signal of the insertion cluster in the genomic vicinity of gene 1 is diluted and may not reach the required significance threshold to be called a CIS. Moreover, gene 2 is not necessarily the actual target gene of the insertion cluster in its neighbourhood. Thus, while searching for candidate target genes, insertions can be associated to the wrong target gene or left without a clear target.
