# Supplementary material for: 3D hotspots of recurrent retroviral insertions reveal long-range interactions with cancer genes
Source: Nat Commun. 2015 Feb 27;6:6381. doi: 10.1038/ncomms7381 (PMC4351571; doi:10.1038/ncomms7381)
Supplement: Supplementary Information — Supplementary Figures 1-17 [file ncomms7381-s1.pdf]

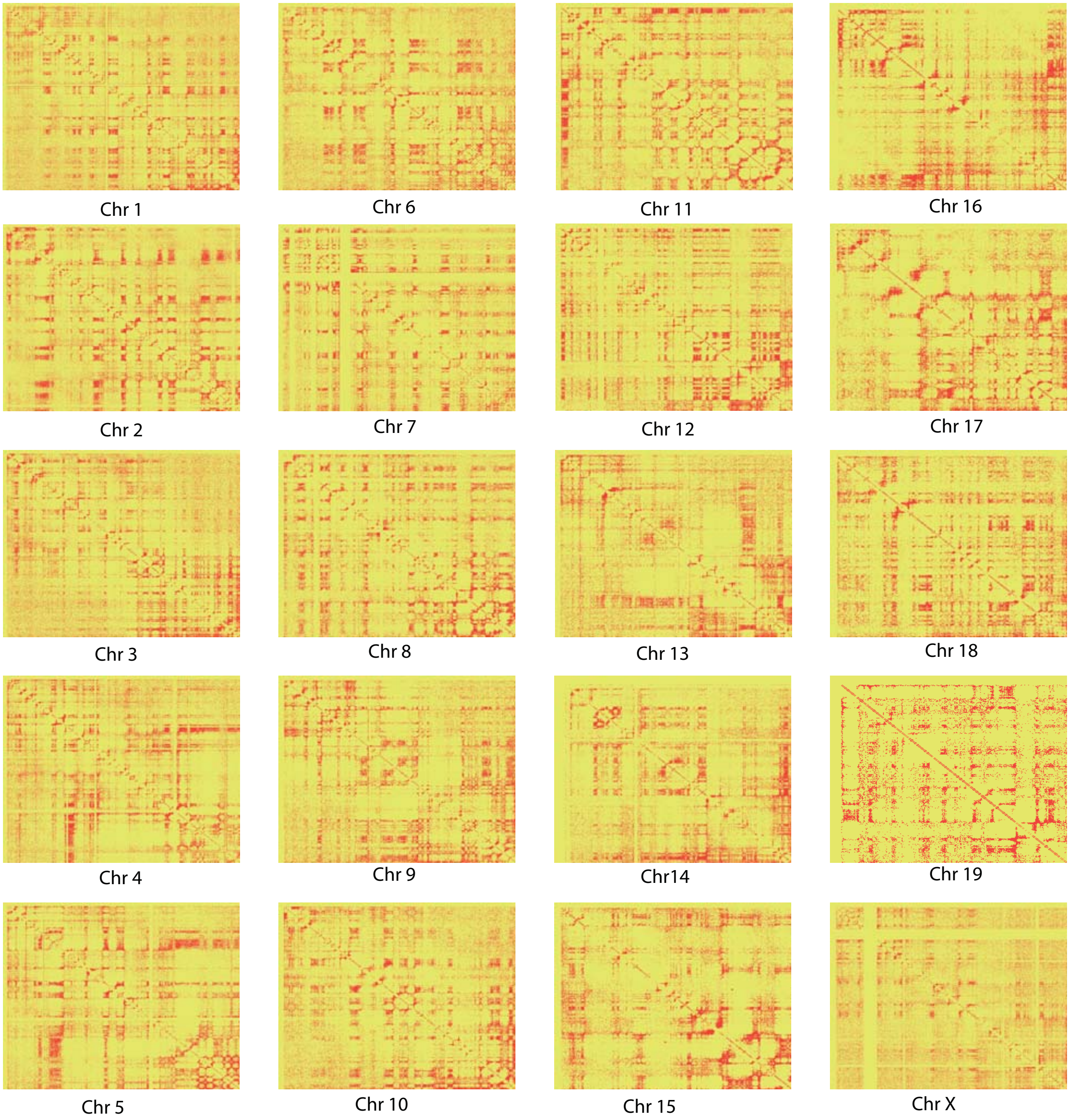

Supplementary Figure 1: **Rank-normalized Hi-C contact matrix for all mouse chromosomes.** The genomic distance bias in the Hi-C contact map is eliminated by using a rank-based normalization (described in the main text). The normalized matrix shows a plaid pattern representing the two-compartment model of chromatin.

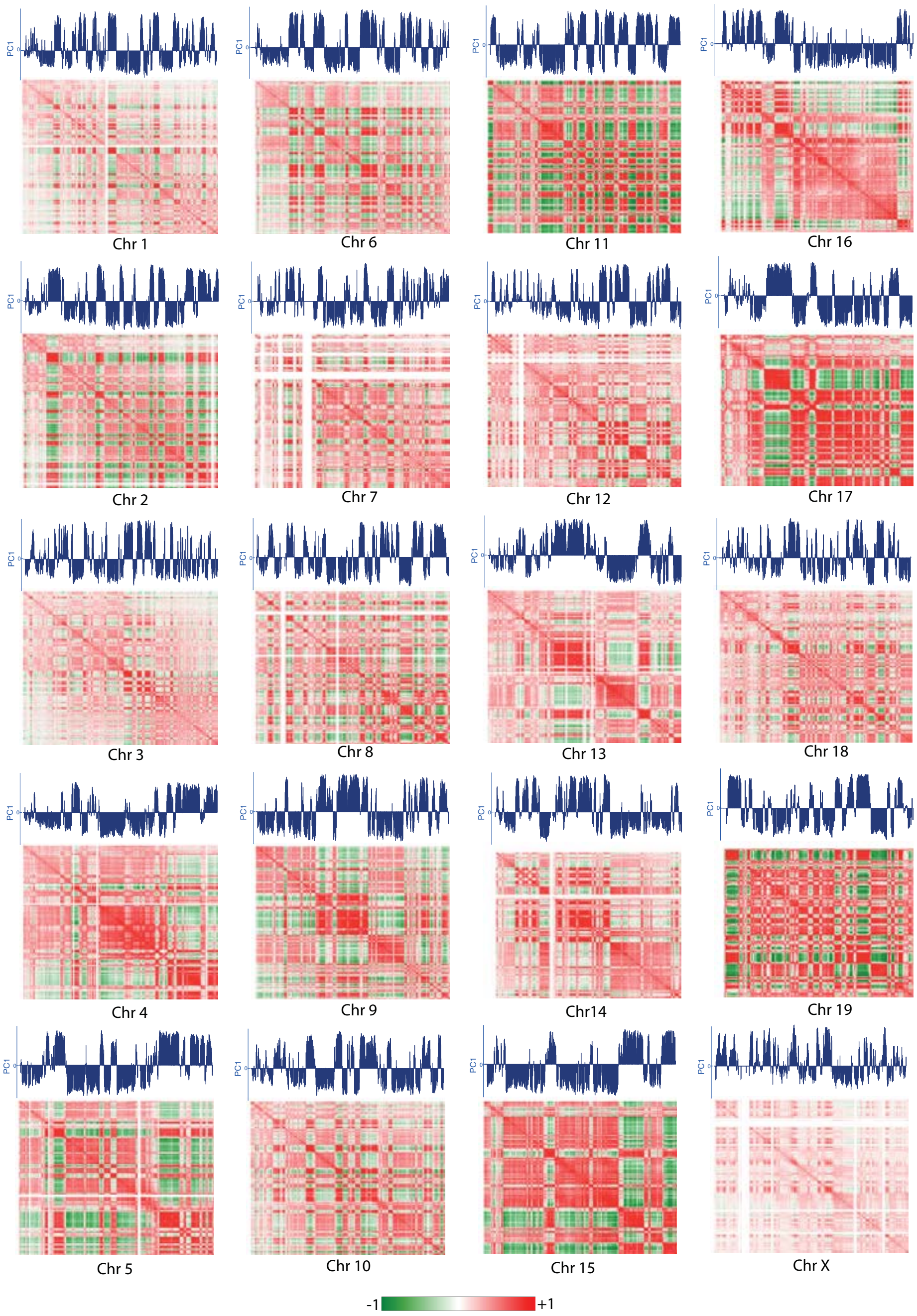

Supplementary Figure 2: **Correlation matrix of the normalized Hi-C matrix.** Correlation matrix of the normalized Hi-C matrix (at 200kb resolution) reflecting chromatin compartmentalization. The open and closed chromatin compartments can be determined based on the sign of the first principle component (PC) of the correlation matrix indicated by the blue curve above each heatmap). The first PC also correlates with chromatin dynamic features such as DNAseI sensitivity, activating histone modifications, repressive histone modifications. Green blocks indicate negative correlation, i.e. Hi-C interactions between open and closed compartments. Red blocks indicate positive correlation, i.e. Hi-C interactions between two open or two closed chromatin compartments.

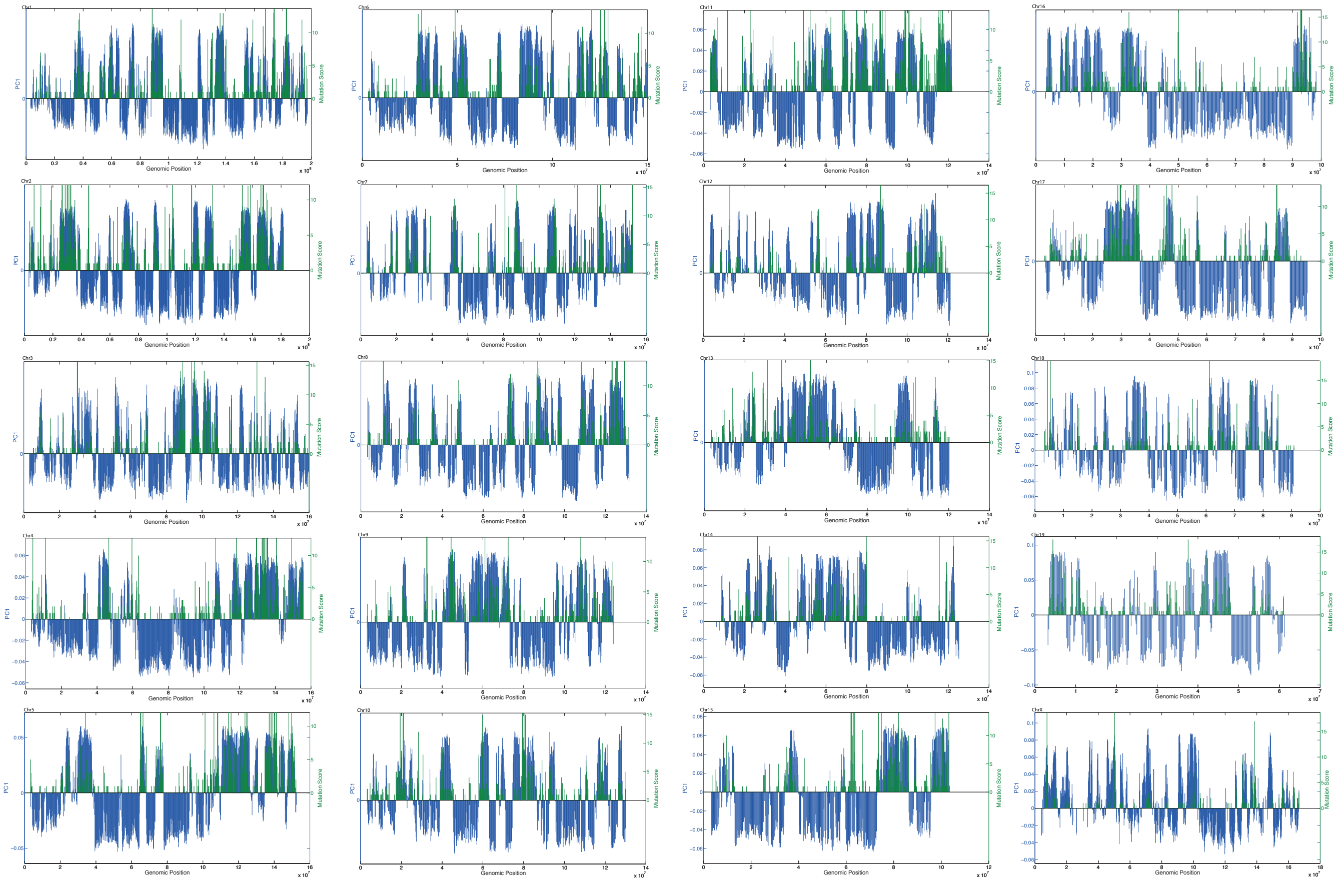

Supplementary Figure 3: **PC1 of correlation matrix of the normalized Hi-C matrix.** The insertion count per bin on each chromosome together with the value of the first principle component (PC1) of the correlation matrix of the normalized Hi-C matrix (at 200kb resolution).Positive and negative value of the PC1 refer to the open and closed chromatin compartments, respectively.

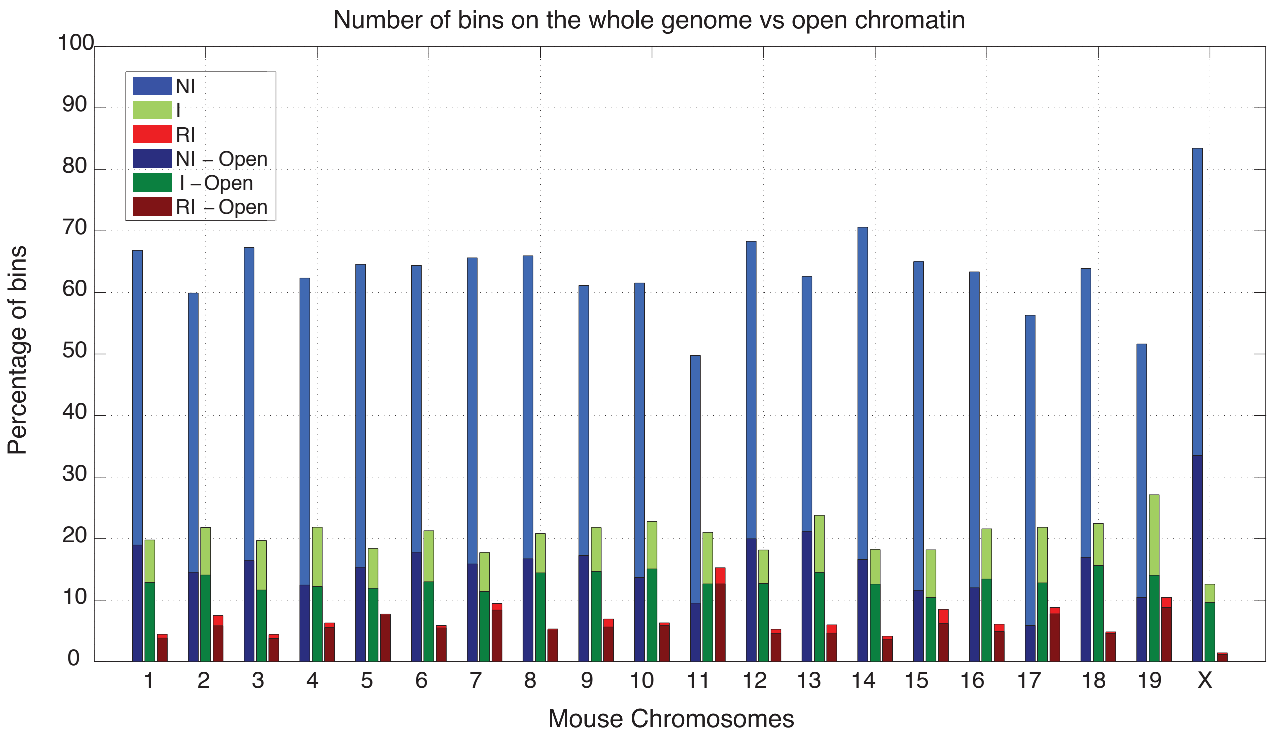

Supplementary Figure 4: **Percentage of bin-pair categories.** Percentage of non-inserted, inserted and recurrently inserted bins along the whole genome as well as within open chromatin compartments. The number of non-inserted bins is decreased in open chromatin. Moreover, most recurrently inserted bins are in open-chromatin.

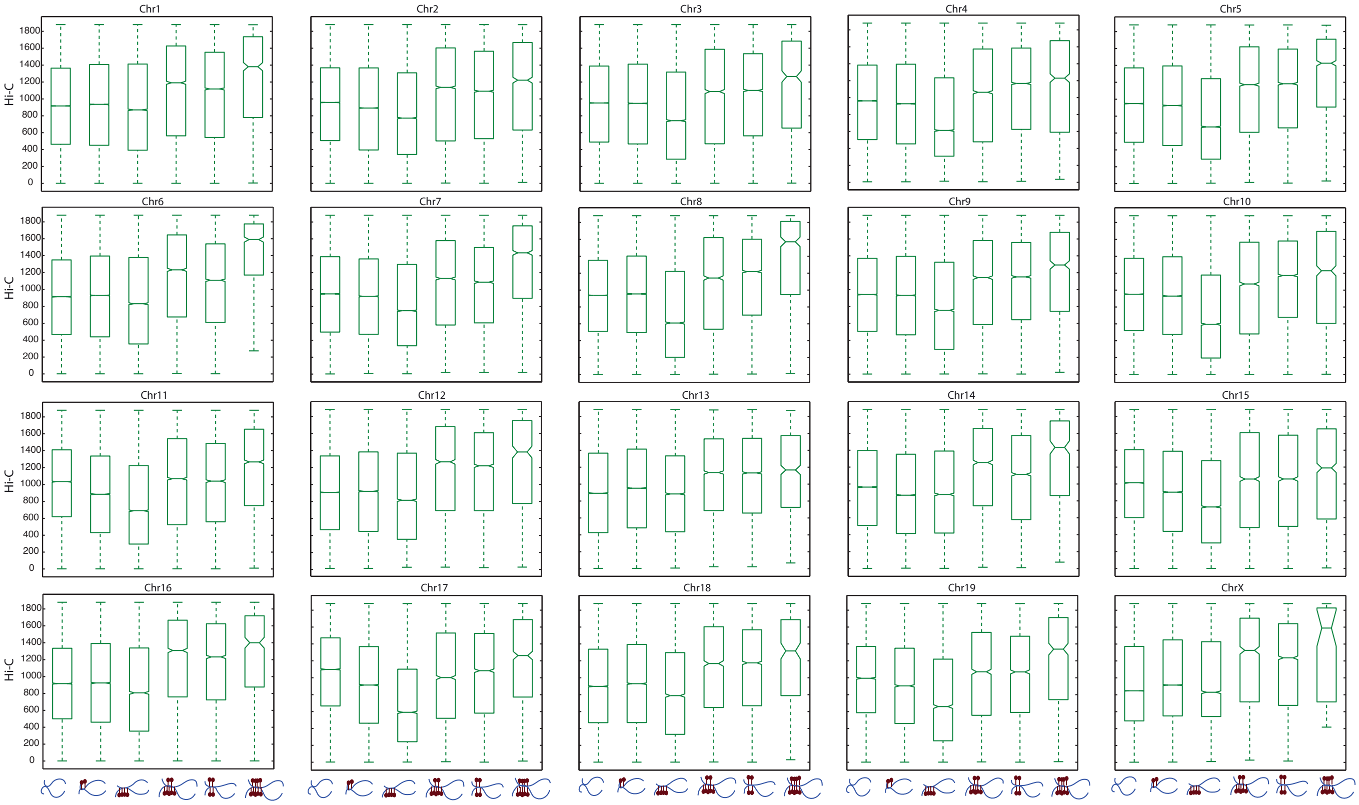

Supplementary Figure 5: **Distribution of rank-normalized Hi-C interactions within ES-cells.** Distribution of Hi-C scores for six bin-pair categories (as described in Fig. 3a with $N_m = 2$ and $N_r = 5$) for Chromosome 1 through X.

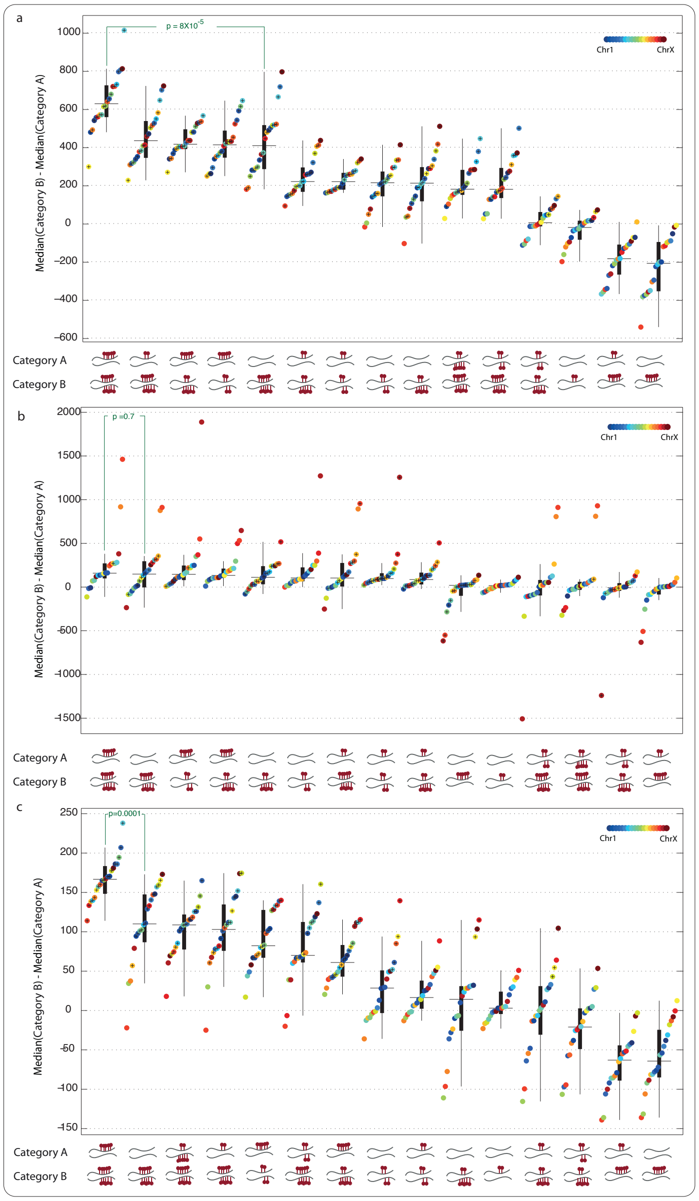

Supplementary Figure 6: **Box plots of spatial associations of inserted loci at different resolutions.** Each box represents a difference between medians for two of the six defined bin-pair classes using bin-size of a) 200kb (also shown in Fig. 3c), b) 40kb and c) 1Mb. A starred circle indicates a significant difference between medians for that chromosome (Wilcoxon rank-sum test; $p-$value $< 10^{-10}$). The y-axis represents the difference between the median Hi-C score of bin-pair category A and B. The bin-pair categories that are compared are schematically illustrated under each box. Boxes are sorted based on their medians. The best distinction between bin-pair classes occurs at 200kb. For bin-size of 200kb, the difference between median Hi-C scores when bin-pair categories A and B represent interactions between RI-NI and RI-RI bin-pairs is significantly higher than the difference between the median Hi-C scores when bin-pair categories A and B represent interactions between NI-NI and RI-RI bin-pairs (paired Wilcoxon rank test; $p-$value $= 8 \times 10^{-5}$).

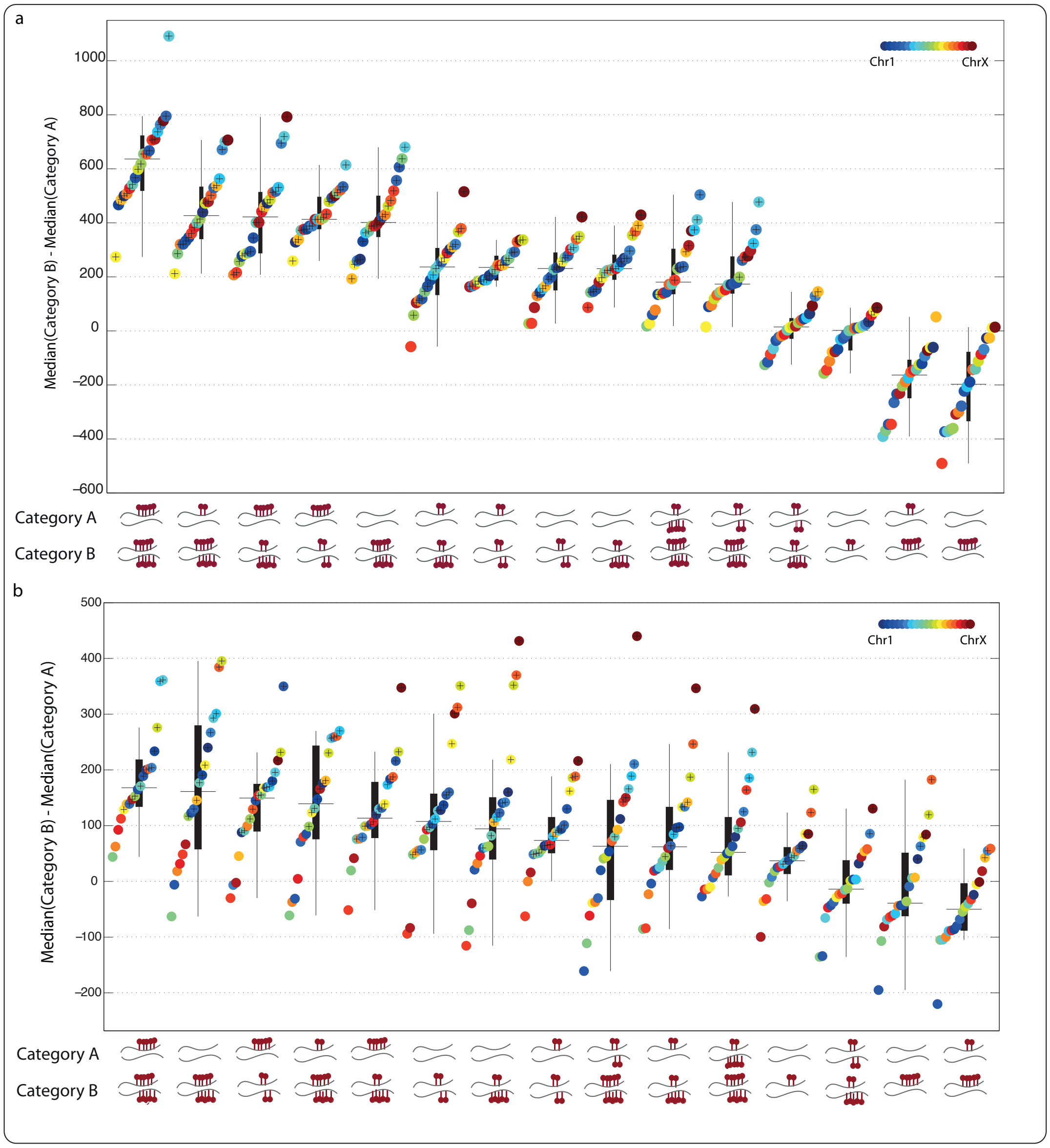

Supplementary Figure 7: **Box plots of spatial associations of inserted loci.** Each box represents a difference between medians for two of the six defined bin-pair classes. a) in bins with at least one transcription start site (TSS-bins), b) in **open** chromatin. We show that, for most chromosomes, genomically distal regions in the genome that carry insertions are more likely to be in spatial proximity in the 3D confirmation of the genome (Fig. 3c). This remains significant if we correct for the fact that integrations are more frequently found in in domains of open chromatin or in genomic vicinity of the TSS of the genes.

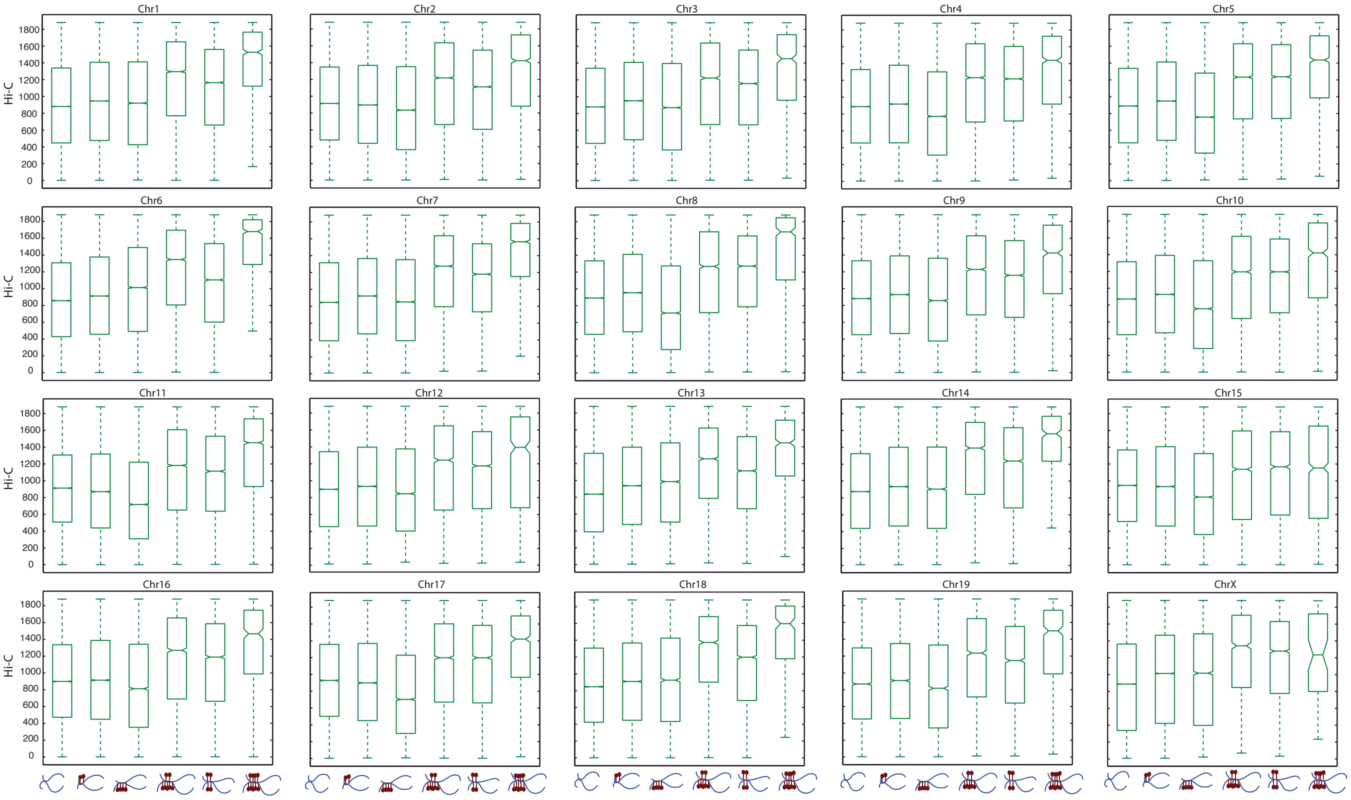

Supplementary Figure 8: **Distribution of rank-normalized Hi-C interactions within cortex-cells.** Distribution of Hi-C scores for six bin-pair categories (as described in Fig. 3 with $N_m = 2$ and $N_r = 5$) for Chromosome 1 through X.

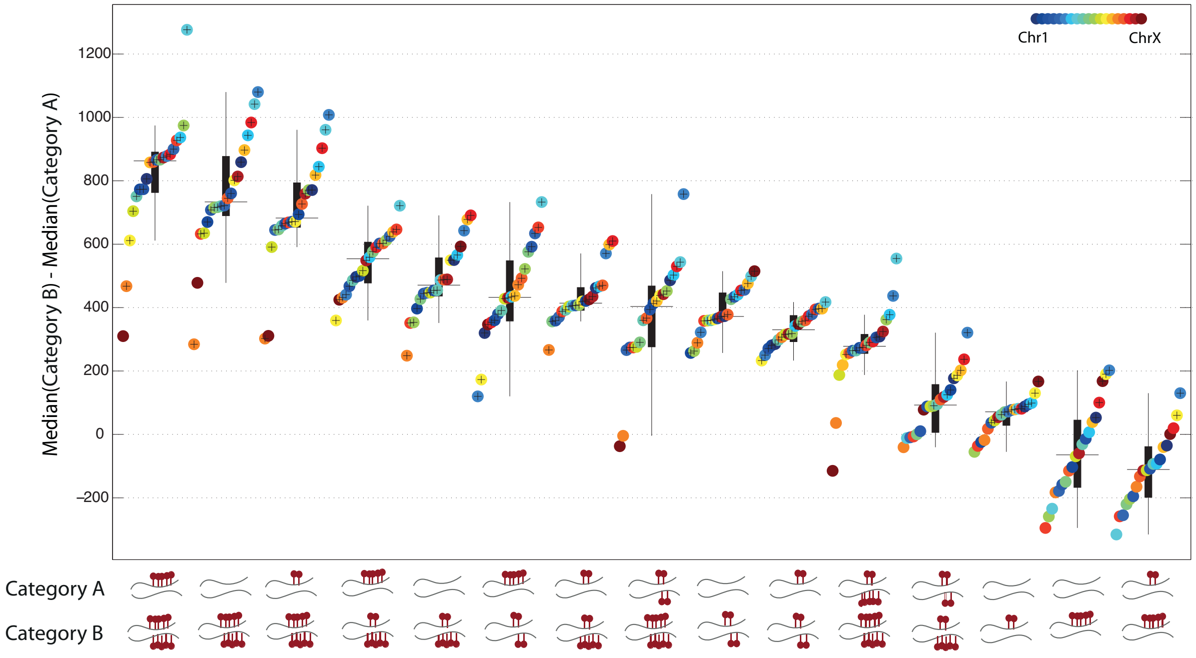

Supplementary Figure 9: **Box plot of spatial associations of inserted loci summarizing Supplementary Fig. 8.** Each box represents the difference between medians of Hi-C distributions of Cortex-cells for two of the six defined bin-pair classes.

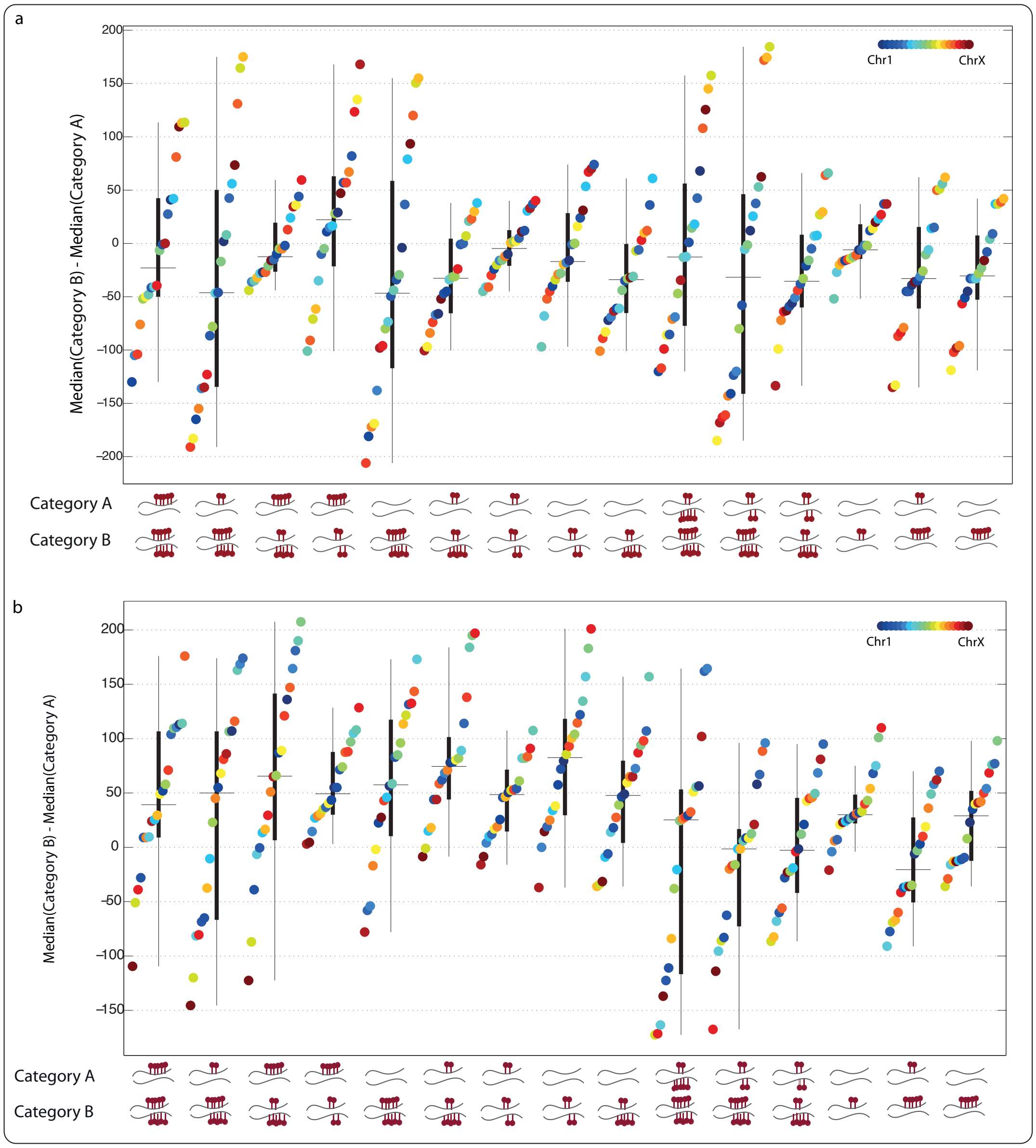

Supplementary Figure 10: **Box plots of spatial associations of randomized mutated loci.** Each box represents a difference between medians for two of the six defined bin-pair classes based on the randomizing the insertion locations a) uniformly across the genome, b) in bins with at least one TSS. Randomizing the insertion locations destroys the observed association.

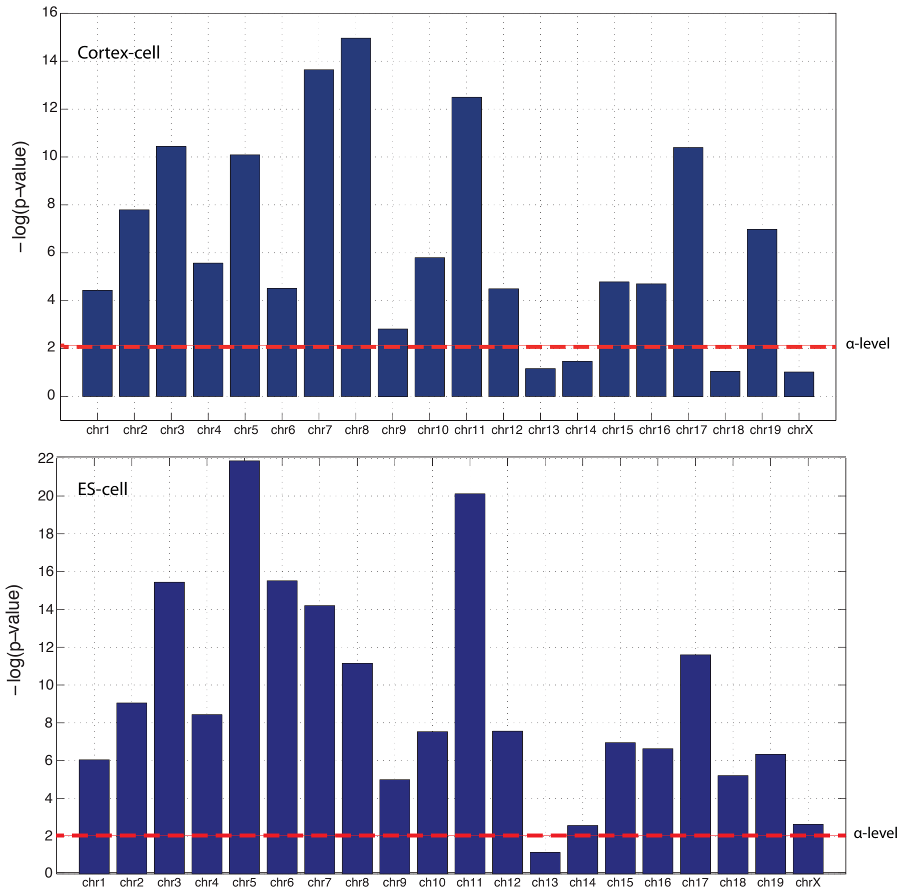

Supplementary Figure 11: **Comparison of mutation scores of nearest spatial neighbors.** Bargraph of $-log(p-$values) obtained by comparing (using the Wilcoxon rank-sum test) the mutation scores of nearest spatial neighbors (NSN) of mutated bins against mutation scores of NSNs of non-mutated bins. The upper panel gives the result for Hi-C data obtained in cortex cells and the lower panel gives the result for Hi-C data obtained in ES-cells. A high $-log(p-$value) indicates that the mutation score of NSNs of mutated regions are significantly higher than mutation scores of NSNs of non-mutated regions. It implies that a mutated bin is significantly more frequently in contact with another mutated bin than non-mutated bins. To define the NSN for a bin, consider a bin of interest b_i. The NSN of b_i (b_j) is found by searching for the maximum Hi-C contact between b_i and all bins along the chromosome. To guarantee that this maximal Hi-C contact is due to the 3D conformation of the genome rather than sequence proximity, the genomic distance between b_i and b_j is required to be more than a given genomic distance d_{NN}.

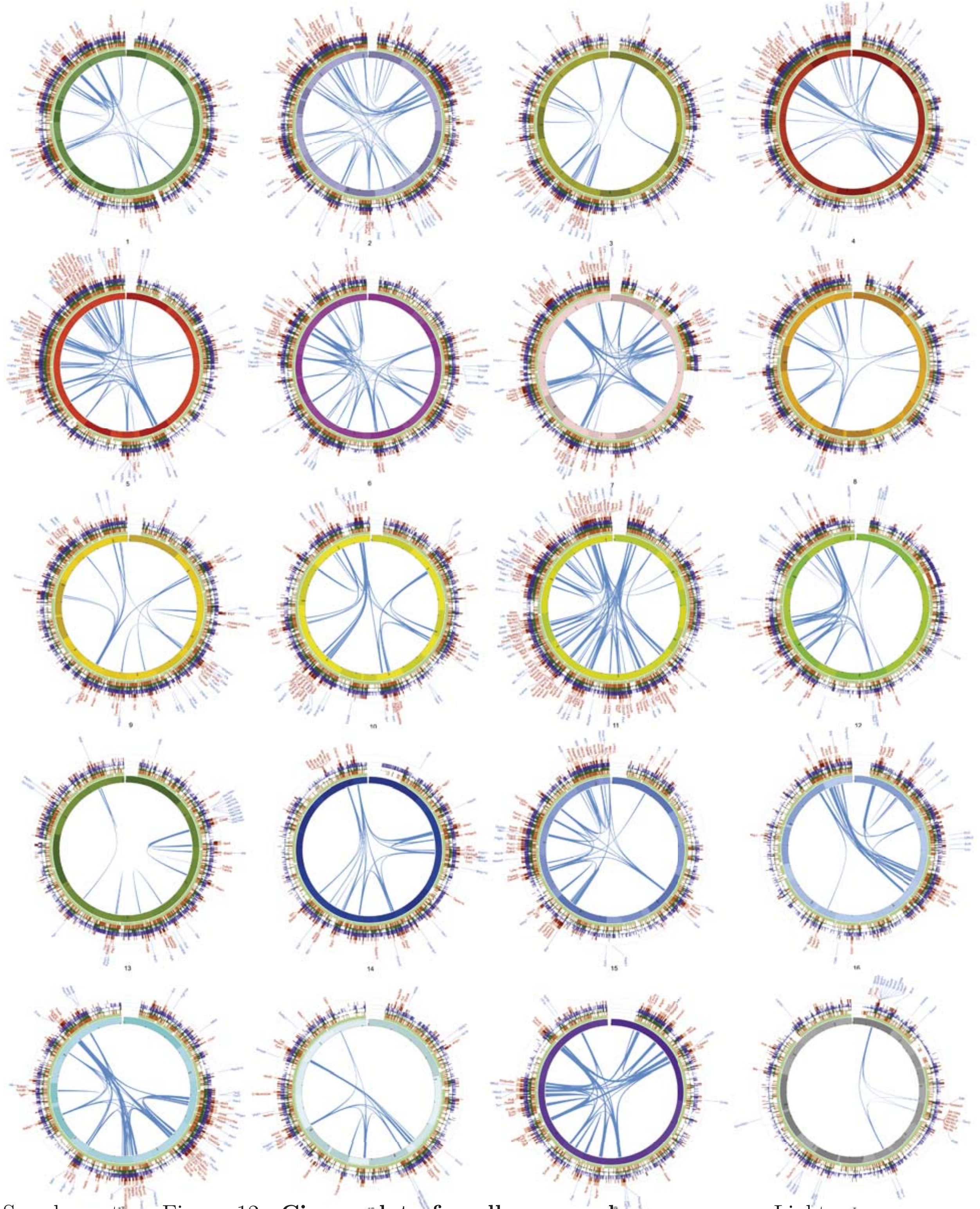

Supplementary Figure 12: **Circos plots for all mouse chromosomes.** Light green, orange, dark green, purple, and red tracks indicate Topologically Associating Domains (TADs), Enhancers, DNase I hypersensitive sites (DHSs),Transcription Factor Binding Sites (TFBSs) for cMyc, CTCF, Taf3, Zfx, Mcaf1, and insertion sites, respectively. Tick marks appear every 5Mb on the chromosome. The ICs are indicated by light red. Links indicate significant Hi-C contacts between ICs. CIS- and CGC-genes are indicated by red and blue, respectively.

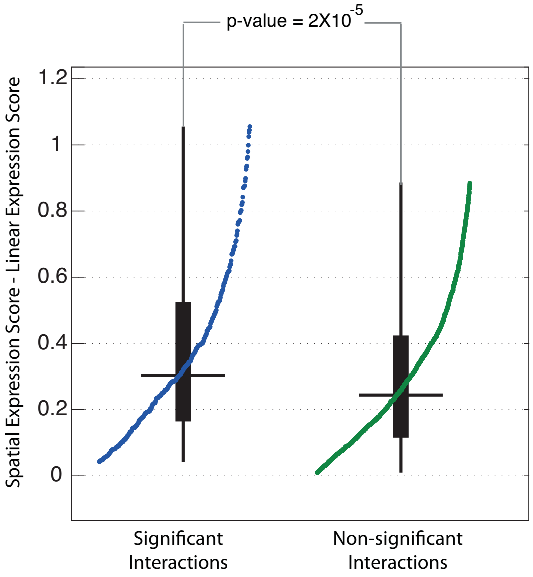

Supplementary Figure 13: **Boxplot of expression association scores.** Expression association scores were calculated for significant CLIC-loci (i.e. the actual CLICs) and non-significant combinations of CLIC-loci. The y-axes represents the difference between spatial and linear expression association scores (defined in the main text) of genes located in a subset of ICs.

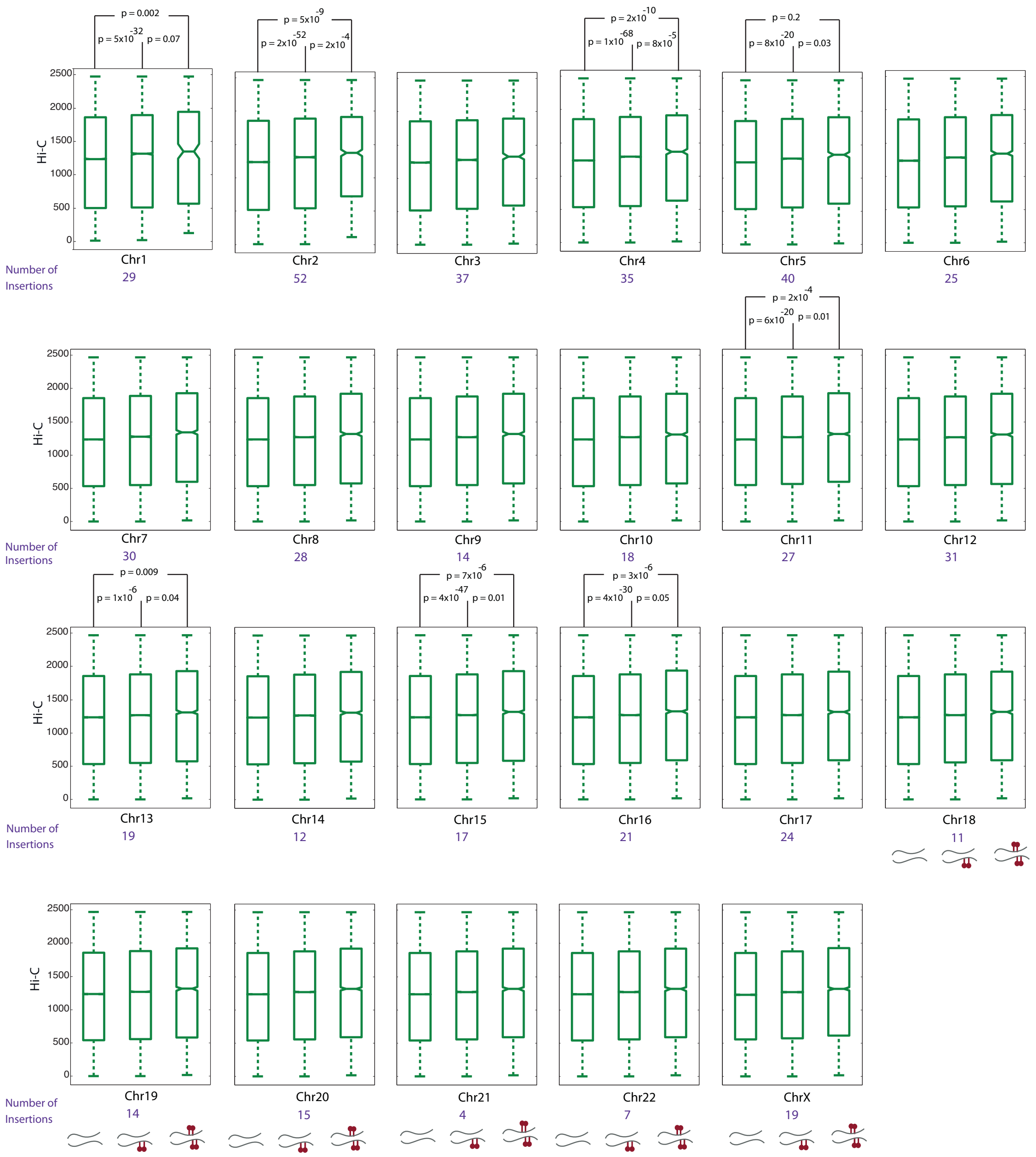

Supplementary Figure 14: **Spatial association between viral insertion sites in the human genome for Hepatitis B virus (HBV) integrations.** Due to the small sample size, we only compared Hi-C contacts between combinations of non-inserted (NI) and inserted (I) bins (3 combinations in total). Comparison of the difference between medians of Hi-C ranks between bin-pairs categories shows that bin-pairs that both harbor insertions are significantly co-localized in terms of the 3D organization of the human genome (Wilcoxon rank-sum test; $p-$value $< 10^{-10}$) in Chromosome 2, 4, 11, 15, and 16. The result is consistent with the co-localization of insertion sites in the mouse genome.

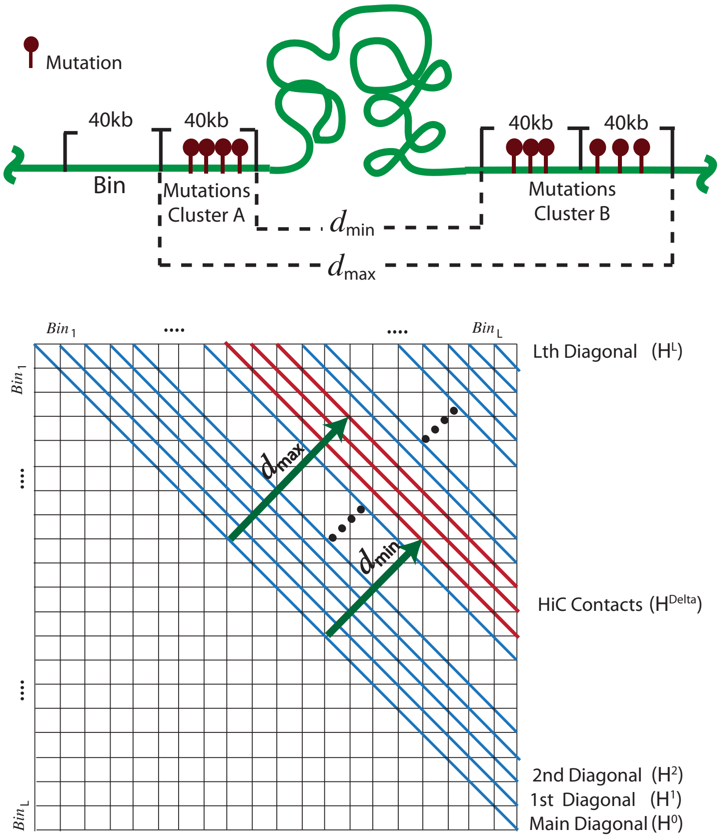

Supplementary Figure 15: **Schematic depiction of the construction the negative distribution for testing co-localization between inserted bins.** The distribution of Hi-C interactions between two clusters of mutations C_A and C_B with size of $n = 2$ and $m = 1$ bins consists of $n \times m$ values (positive distribution). A negative distribution is generated by concatenating superdiagonals in the Hi-C matrix. These superdiagonals contain the Hi-C contact frequencies between bins with genomic distances in range of $\delta = [d_{min},\ d_{max}]$ where d_{min} and d_{max} are calculated based on the maximum and minimum genomic distance between C_A and C_B. This allows for an hypothesis test to assess if the distribution of Hi-C values between the pair of inserted bins is significantly different from pairs of bins with similar genomic distance. Note that to guarantee that there is a sufficient number of values in the positive distribution we set the minimum size of an IC to three bins so that each positive distribution consists of at least $3 \times 3 = 9$ points. To this end, ICs with size of less than three bins are expanded to cover one more bin on both sides. This is not depicted in the figure.

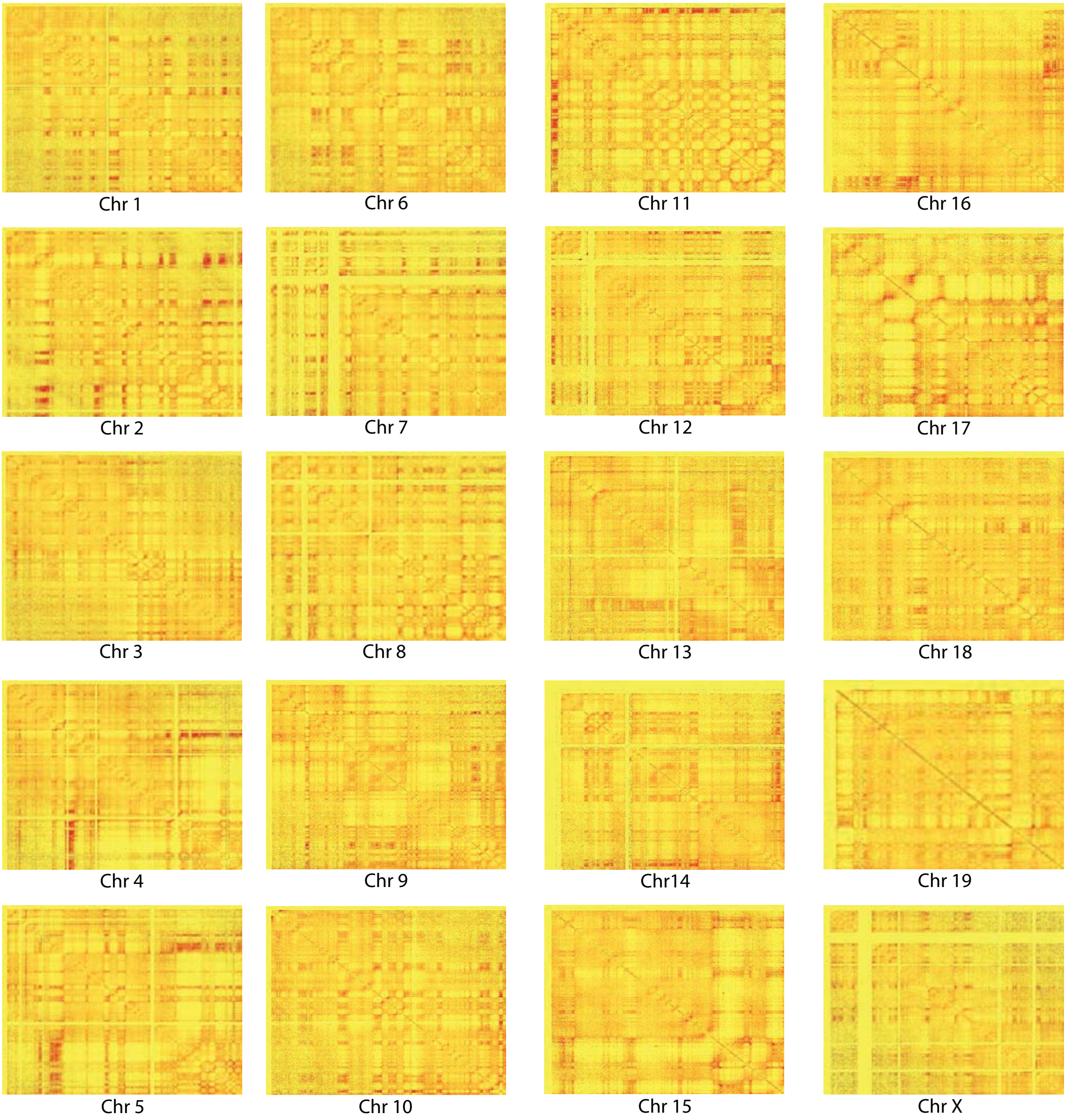

Supplementary Figure 16: **Normalized Hi-C contact matrix for all mouse chromosomes using average-based normalization method.** The genomic distance bias in the Hi-C contact map is eliminated by dividing each entry in the Hi-C contact matrix by the genome-wide average of Hi-C contacts between regions at the same genomic distance. Similar to the rank-normalized matrix, the normalized matrix shows a plaid pattern pointing to a two-compartment model of chromatin.

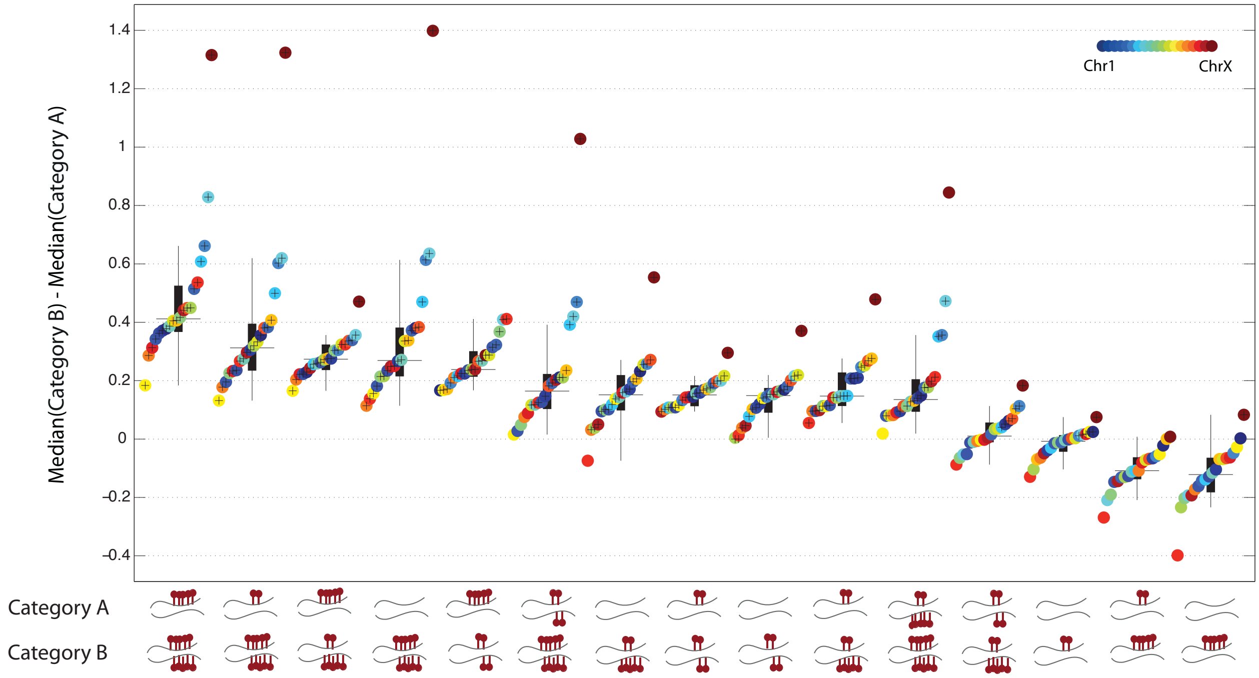

Supplementary Figure 17: **Box plot of spatial associations of inserted loci using average-based normalization method.** Each box represents a difference between medians for two of the six defined bin-pair classes using the average-based normalization. We show that, for most chromosomes, genomically distal regions in the genome that carry insertions are more likely to be in spatial proximity in the 3D confirmation of the genome using the rank-normalized Hi-C matrix. This is also significant if we used this normalization method.
